# Multishot versus Single-Shot Pulse Sequences in Very High Field fMRI: A Comparison Using Retinotopic Mapping

**DOI:** 10.1371/journal.pone.0034626

**Published:** 2012-04-13

**Authors:** Jascha D. Swisher, John A. Sexton, J. Christopher Gatenby, John C. Gore, Frank Tong

**Affiliations:** 1 Department of Psychology and Vision Research Center, Vanderbilt University, Nashville, Tennessee, United States of America; 2 Department of Biomedical Engineering, Vanderbilt University, Nashville, Tennessee, United States of America; 3 Institute of Imaging Science, Vanderbilt University, Nashville, Tennessee, United States of America; 4 Department of Radiology and Radiological Sciences, Vanderbilt University, Nashville, Tennessee, United States of America; University of Leuven, Belgium

## Abstract

High-resolution functional MRI is a leading application for very high field (7 Tesla) human MR imaging. Though higher field strengths promise improvements in signal-to-noise ratios (SNR) and BOLD contrast relative to fMRI at 3 Tesla, these benefits may be partially offset by accompanying increases in geometric distortion and other off-resonance effects. Such effects may be especially pronounced with the single-shot EPI pulse sequences typically used for fMRI at standard field strengths. As an alternative, one might consider multishot pulse sequences, which may lead to somewhat lower temporal SNR than standard EPI, but which are also often substantially less susceptible to off-resonance effects. Here we consider retinotopic mapping of human visual cortex as a practical test case by which to compare examples of these sequence types for high-resolution fMRI at 7 Tesla. We performed polar angle retinotopic mapping at each of 3 isotropic resolutions (2.0, 1.7, and 1.1 mm) using both accelerated single-shot 2D EPI and accelerated multishot 3D gradient-echo pulse sequences. We found that single-shot EPI indeed led to greater temporal SNR and contrast-to-noise ratios (CNR) than the multishot sequences. However, additional distortion correction in postprocessing was required in order to fully realize these advantages, particularly at higher resolutions. The retinotopic maps produced by both sequence types were qualitatively comparable, and showed equivalent test/retest reliability. Thus, when surface-based analyses are planned, or in other circumstances where geometric distortion is of particular concern, multishot pulse sequences could provide a viable alternative to single-shot EPI.

## Introduction

Much of the promise of very high field (> 3T) MR imaging stems from the improvements in signal-to-noise (SNR) that are expected to result from increased magnetic field strength. Greater field strength results in higher signal intensity; however, this increased intensity produces not only stronger task related BOLD activation but also magnifies physiological noise. This enhancement of physiological noise may limit the SNR gains achievable through very high field fMRI at standard (∼3 mm) resolutions [1], [2]. However at higher resolutions, a proportionately larger role is played by non-physiological intrinsic noise sources [2], [3], which are not expected to be enhanced to the same degree by increasing field strength [1], [4]. Thus as spatial resolution increases, very high field imaging offers correspondingly greater benefits [3]. High-resolution fMRI is thus widely considered to be a promising application of very high field MRI.

Despite this potential, an array of technical challenges must be addressed before the likely benefits of very high field imaging can be easily accessed by a wide range of investigators. One such issue that becomes more salient at very high field strengths is that of geometric distortion. The vast majority of functional MRI performed at field strengths of 3T and below uses single-shot 2D echo-planar imaging (EPI), in which the k-space representation of an excited slice is read out in a single extended echo train. These extended readout periods make EPI sequences very efficient in terms of SNR per unit time, and thus highly sensitive to functional activation. Single-shot EPI therefore became widely used for fMRI as soon as gradient hardware capable of supporting its demanding requirements was commonly available, largely displacing earlier multishot gradient-echo alternatives such as FLASH (fast low angle shot) functional imaging [5].

One price paid in return for the temporal efficiency of single-shot EPI is that its extended readouts also make it more susceptible to off-resonance effects, of which geometric distortion is one example. “Off-resonance” refers to the rate of precession of excited spins, which can be shifted off of the expected resonant frequency by uncompensated spatial gradients in the magnetic field. In addition to large-scale background gradients, which can often be largely corrected through appropriate shimming, off-resonance conditions can also arise at the interface of materials with differing magnetic susceptibilities, such as air and tissue. These off-resonsance effects can lead to dephasing and signal dropout, as well as geometric distortion. Gradients in the slice direction are particularly prone to causing dropout; in-plane background gradients add to the effects of the imaging gradients, and thus result in dropout only if they are so strong as to shift the signal outside of the acquired k-space region entirely. In-plane gradients are more likely to lead to geometric distortion. An offset of the mean magnetic field strength within a voxel, as would result from local susceptibility gradients, causes a change in the average resonant frequency of the spins within that voxel. This shifted frequency results in phase errors that accumulate over the readout period. Frequency shifts are typically small compared to EPI’s high bandwidth along the frequency encode axis, and thus geometric distortion in EPI is usually considered to be negligible in this direction. The effective bandwidth per pixel is much lower along the phase encode axis, however, leading to potentially large positional shifts in the phase encode direction. Large-scale in-plane background gradients in the readout direction cause shearing of the image in the phase-encode direction, while gradients along the phase encode direction result in scaling (stretching or shrinking) of the image. Such in-plane gradients also change the effective TE, contributing to a loss of BOLD sensitivity [6].

The problem of geometric distortion is exacerbated in very high field, high-resolution imaging. A fixed difference in magnetic susceptibility leads to greater local changes in the magnetic field when the applied field strength is increased, resulting in larger magnitudes of distortion at high field strength, all other factors being equal. Distortion also increases, relative to the size of a single voxel, at higher resolutions. Levels of distortion that could be considered negligible at lower resolutions may therefore become increasingly salient in high-resolution imaging.

Controlling geometric distortion is thus of great interest in high field fMRI. Perhaps the most commonly used strategy to correct for geometric distortion in EPI imaging is field-map-based dewarping [7]–[10]. In these methods, a map of the B0 field inhomogeneities is first calculated from the phase difference between images acquired at separate echo times. The shift in position to be expected from the measured field inhomogeneities can then be determined at each point in the image. A “dewarped” functional image is then generated by applying the reverse shift to the functional data and interpolating back into a regularly spaced grid. This method has been shown to lead to substantial improvements in functional/structural coregistration, provided that subject head movements are modest and the field map itself is sufficiently regularized through spatial smoothing or fitting with low-order basis functions [11], [12].

Distortion can also be controlled at the time of image acquisition. Imaging strategies involving faster readouts (or, equivalently, greater effective bandwidth per pixel in the phase encode direction, or shorter effective echo spacing) reduce the effects of geometric distortion, as off-resonance phase errors have less time in which to accumulate. Increasing the strength of the applied gradients leads directly to faster traversal of k space and therefore lower distortion, limited only by the available hardware or the possibility of subject nerve stimulation. Parallel acceleration [13]–[16], such as via sensitivity encoding (SENSE) [15], enables shorter total readout durations through skipping lines of k space during the initial traversal, with the missing data inferred from the coil sensitivity profiles. R-fold parallel acceleration thereby reduces the magnitude of distortion by the same factor of R. The level of parallel acceleration which can be applied is fundamentally limited only by the number of distinct coil elements, but in practice loss of signal to noise at higher acceleration factors, stemming from the non-independence of nearby coils, limits acceleration to substantially lower levels. Using coil arrays with 8 to 32 elements, as are currently widely available, acceleration factors of 2 to 4 appear readily achievable. In many cases, this combination of high applied gradients and parallel acceleration may suffice to limit distortion in single-shot EPI to acceptable levels, even at very high field strengths [17]–[20].

If controlling distortion is a priority, then in addition to correction through postprocessing and choosing sequence parameters so as to minimize its effects, one may also consider using pulse sequences less inherently susceptible to distortion than conventional single-shot EPI. Though the efficiency of single-shot EPI makes it the dominant class of pulse sequence for most functional imaging applications, in some cases, particularly at very high resolutions, multishot acquisitions represent a viable alternative. In these sequences a single image is reconstructed from k space data acquired separately after two or more RF excitations, or shots. The readout duration after each shot can be made much shorter than in single-shot EPI, as only a fraction of the totality of k space must be traversed. The strategy of dividing a standard 2D EPI readout into multiple segments in this way is referred to as segmented EPI. A principal advantage of this approach is that the achievable spatial resolution is no longer limited by the desired echo time, since the readout of a single segment can be made almost arbitrarily brief. For this reason, segmented EPI is often used for very high-resolution (submillimeter) functional MRI. However in addition to increasing the practically achievable spatial resolution, segmented readouts can also lead to greatly reduced distortion relatively to single-shot EPI. Since the accumulated phase errors from background field gradients are effectively “reset” at each excitation, dividing the total k space traversal into multiple segments also increases the effective readout bandwidth proportionally to the number of shots. On the other hand, a significant disadvantage of multishot imaging is the sensitivity of these sequences to shot-to-shot instabilities, for instance due to head motion or respiration, or instabilities in the instrumentation. Multishot imaging may then be expected to show reduced temporal SNR when compared to similar single-shot sequences [21]–[24].

Despite this lower expected tSNR, segmented acquisitions may be favored in some circumstances because of their lower expected distortion, even at spatial resolutions where single-shot EPI remains feasible. One situation in which this may be the case is when high-resolution surface-based analyses are planned, as a high degree of spatial precision is required to ensure the validity of the analysis. Here, a representation of the cortical surface is first extracted from a subject’s own anatomical images, and functional activation patterns or time series data is then projected onto this reconstructed surface. At higher resolutions, uncorrected geometric distortion is increasingly likely either to shift functional activity off of the cortical reconstruction entirely, making such activity effectively invisible to surface-based analyses; or, if less severe, to substantially disrupt the pattern of activation visualized on the surface. In these circumstances it may prove worthwhile to minimize distortion at the outset by opting for segmented acquisitions, thereby preserving the validity of a subsequent surface-based analysis, even at the cost of some sensitivity to functional activation.

Retinotopic mapping is a canonical example of surface-based fMRI data analysis. The topographic representations of the visual field within human visual cortex can be revealed through the use of relatively standardized mapping stimuli and analysis procedures [25]–[27]. Though the convoluted folding of cortical gyri and sulci makes the organization of visual cortex difficult to visualize in the imaging volume, when projected onto inflated or flattened representations of the cortical surface the arrangement of the visual areas becomes comparatively clear. Whereas in conventional fMRI studies the statistical significance of the activity is typically of greatest interest, in retinotopic mapping one is usually more concerned with the position within the visual field represented by a voxel. This retinotopic position is represented by a pseudocolor overlay, and is expected to vary continuously with changing position on the cortical surface. Since the pattern of the retinotopic maps within the early visual areas is expected to be very regular and stereotyped across subjects, the qualitative impact of uncorrected geometric distortions may be more readily apparent.

For these reasons, we used retinotopic mapping as a test case in which to compare two particular strategies for managing geometric distortion in high field (7T) fMRI. In the first approach, we used parallel-accelerated single-shot EPI followed by field-map-based dewarping. EPI pulse sequences are expected to show good functional sensitivity, but may suffer from residual uncorrected distortion that could impact the resulting retinotopic maps. As an alternative, we investigated the use of multishot 3D gradient-echo sequences (termed “fast field echo”, or FFE [28], [29]), which use shorter echo trains and multiple RF excitations to cover the same region of k space as their single-shot counterparts, along with additional phase encoding steps in the slice (slab) select direction. The repeated RF excitations in the multishot sequences lead to higher effective bandwidths in the phase-encode direction than the equivalent single-shot sequences (by roughly a factor of 3 for the sequences used here), and therefore a reduction by the same factor in the expected magnitude of distortion. Because of shot-to-shot phase instabilities stemming from physiological processes or other factors, however, the multishot FFE sequences may have worse temporal SNR than single-shot EPI. We compared these strategies across three different spatial resolutions (1.1, 1.7, and 2.0 mm isotropic), as the relative significance of geometric distortions, and thus the tradeoffs required to successfully manage it, are expected to vary across resolutions.

These acquisition strategies represent two relatively dissimilar approaches to fMR imaging. As a consequence, it is difficult to attribute differences in outcome to any one critical factor that differs between the sequences tested. However, such a choice between disparate acquisition strategies mirrors that faced in practice by other investigators attempting to take advantage of high field fMRI. It is to be hoped that these observations can serve as a practical, useful point of reference, and an illustration of some of the tradeoffs inherent in optimizing strategies for functional imaging.

## Results

We imaged 6 participants as they viewed rotating, counterphasing checkerboard wedge stimuli, as are typically used for polar angle retinotopic mapping [25]–[27]. The participants were scanned using two different types of imaging sequence, accelerated single-shot EPI and accelerated multishot 3D-FFE, at three different isotropic resolutions (1.1, 1.7, and 2.0 mm - see Table 1 for sequence parameters). Total imaging time was matched across sequences and resolutions, with two 288 s functional runs completed at each sequence/resolution combination. The effective bandwidth of the FFE sequences was roughly a factor of 3 greater than that of the EPI sequences at the same resolution, and therefore the FFE images were in general expected to experience roughly 3 times less geometric distortion than their EPI counterparts. At 2.0 mm resolution, the readout period of the FFE sequence was sufficiently brief so that echo-shifting [30] could be applied, resulting in a 3D-PRESTO (PRinciple of Echo-Shifting with a Train of Observations) sequence [31], [32]. This allowed for a considerable reduction in the time required to acquire a single functional image (see methods).

**Table 1:** Sequence parameters.

Voxel Size	FOV	Matrix	Sequence	T*_acq_*	TR	TE	Flip	ETL	R*_PE_*	BW*_PE_*
1.1 mm	144 × 144 × 28 mm	128	2D-EPI	2.88 s	2880 ms	22 ms	80°	55	2.4	21.2 Hz/pix
1.1 mm	144 × 144 x 28 mm	128	3D-FFE	4.0 s	42 ms	22 ms	20°	17	2.6	62.7 Hz/pix
1.7 mm	160 × 160 × 40 mm	96	2D-EPI	2.88 s	2880 ms	22 ms	80°	37	2.7	41.3 Hz/pix
1.7 mm	160 × 160 × 40 mm	96	3D-FFE	2.88 s	31 ms	22 ms	10°	13	2.6	101.3 Hz/pix
2.0 mm	160 × 160 × 40 mm	80	2D-EPI	2.4 s	2400 ms	22 ms	80°	31	2.7	55.2 Hz/pix
2.0 mm	160 × 160 × 40 mm	80	3D-PRESTO	1.5 s	19 ms	26 ms	10°	13	2.2	114.1 Hz/pix

Abbreviations: FOV: field of view; T*_acq_*: volume acquisition time; TR: repetition time; TE: echo time; ETL: echo train length; R*_PE_*: parallel acceleration factor (R) in the ky phase encode direction; BW*_PE_*: sequence bandwidth in the ky phase encode direction.

B0 field maps showed that the standard deviation of the resonant frequency within V1 grey matter voxels was 38 Hz on average (+/– 17 Hz s.d. across subjects). We calculated the corresponding physical displacement expected for the 1.1 mm EPI sequence, which had the lowest effective bandwidth per millimeter in the phase encode direction of all the tested sequences, and which was therefore expected to show the greatest physical magnitude of distortion. An expected physical displacement (in millimeters) was calculated on the basis of the field map for each V1 voxel, assuming an effective bandwidth of 21.2 Hz/pixel and pixel size of 1.1 mm, as in the high-resolution EPI sequence. For this sequence, the median expected absolute positional displacement across the V1 voxels averaged 1.04 mm across subjects.

Standard techniques were used to reconstruct the cortical visual field maps from the functional data [26] (see methods). Briefly, voxels were deemed to be significantly active if their time series showed significantly greater modulation at the temporal frequency of the rotating visual stimulus than at other “noise” frequencies. These active voxels were then rendered using a color map indicating the phase of the time series Fourier component at the stimulus frequency. This response phase indicates the angular position of the wedge at the time of the voxels’ peak response, with a constant temporal offset reflecting hemodynamic lag. Thus response phase can be taken to represent the angular position within the visual field to which a voxel is most sensitive.

**Figure 1:**
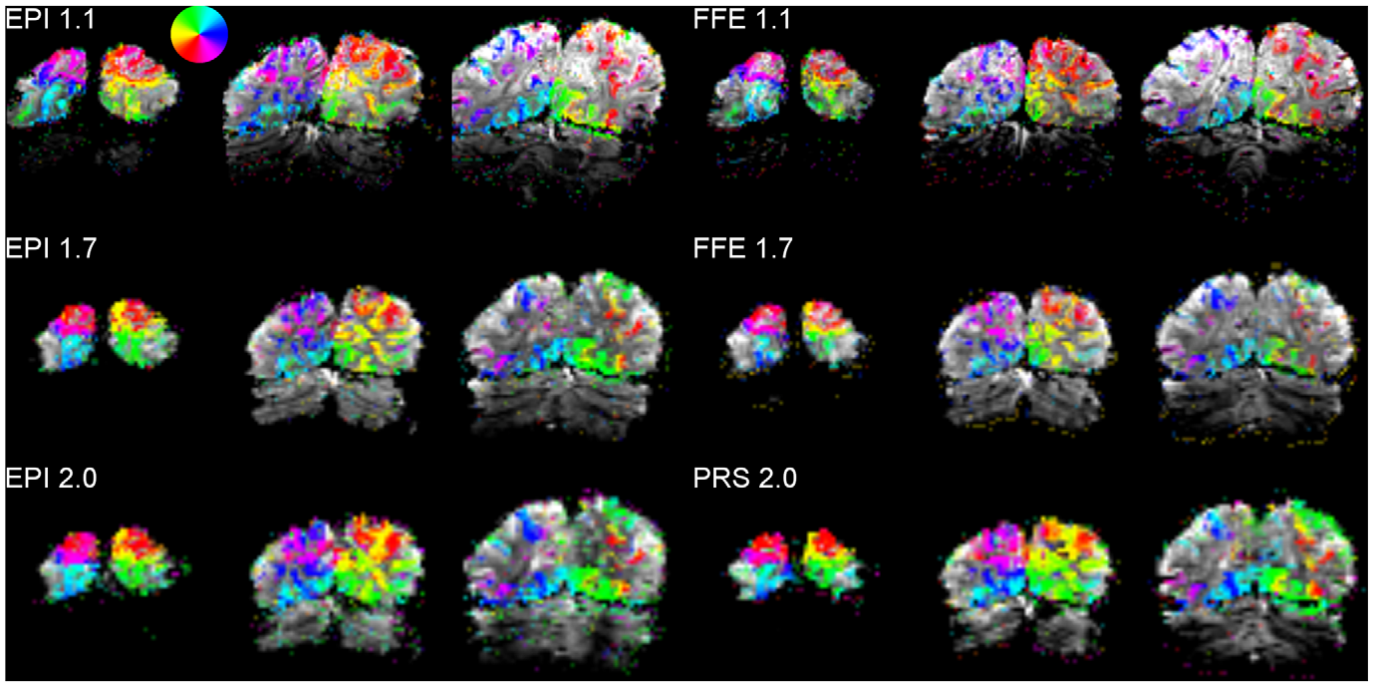
Retinotopic activation overlaid on mean functional images. Voxels rendered in color are significantly active at levels from *p*<0.01 (translucent) to *p*<10^-5^ (opaque), with color indicating response phase (see inset color key). Images are displayed in neurological convention. PRS: 3D-PRESTO.

In order to inspect the quality of the functional images and activation, the retinotopic maps were first plotted as slices within the imaged brain volume (Fig. 1). The multishot and single-shot sequences both yielded broadly comparable activation patterns, with both showing activation largely restricted to the cortical grey matter even at a relatively lenient statistical threshold of *p*<0.01 (uncorrected). Some degree of geometric distortion was evident in the single-shot EPI images for several participants, manifesting as skew along the right/left (phase encode) dimension that produced a visually salient apparent asymmetry between the right and left hemispheres. Such distortion was less evident in the multishot FFE images.

**Figure 2:**
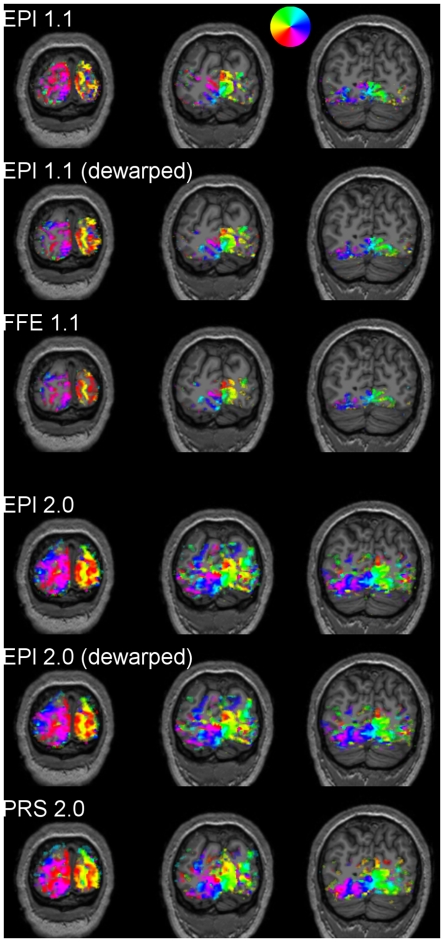
Retinotopic activation maps resampled and overlaid on T1-weighted anatomical images. Voxel significance and color map are as in the previous figure.

To help gain a qualitative appreciation for the potential effects of these levels of geometric distortion on subsequent surface-based analyses, the volumetric functional activation maps were resampled and aligned with each participant’s T1-weighted anatomical volume, acquired previously at 3T with 1 mm^3^ spatial resolution (Fig. 2). As anatomical images are only minimally susceptible to inhomogeneity-induced geometric distortion, they provide a good “ground truth” against which to evaluate the relative distortion within the functional volumes. The functional and anatomical images were aligned by an automated boundary-based registration algorithm, which attempts to position the functional image so as to maximize the difference in image intensity across the anatomically-defined grey matter/white matter border [33]. Reasonably good global alignment between the functional and anatomical images could be achieved for both the single- and the multishot functional data, though the greater distortion within the single-shot images resulted in subtle but noticeable local misalignments in some participants, particularly on the lateral surfaces of the brain. These local misalignments were more readily apparent in the higher-resolution functional data. Field-map-based dewarping [8] of the high-resolution single-shot statistical activation maps led to qualitatively appreciable improvements in their alignment with the anatomical volumes.

**Figure 3:**
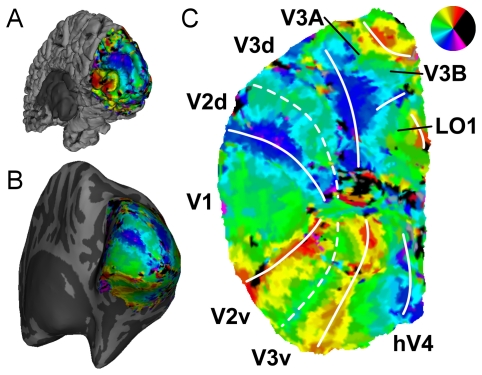
Retinotopic maps on the cortical surface. A map of response phase in the right hemisphere is shown on the folded pial surface of cortex (A), on the inflated hemisphere (B), and on a flattened representation of the occipital lobe with areal borders overlaid (C). Color represents responses to stimuli in the contralateral visual field (see inset). The retinotopic maps are largely occluded by the folded cortical surface, but can be more easily viewed on the flattened occipital patch. Borders are defined following [36]. Data shown are from Subject 1, using the 2.0 mm 3D-PRESTO sequence.

**Figure 4:**
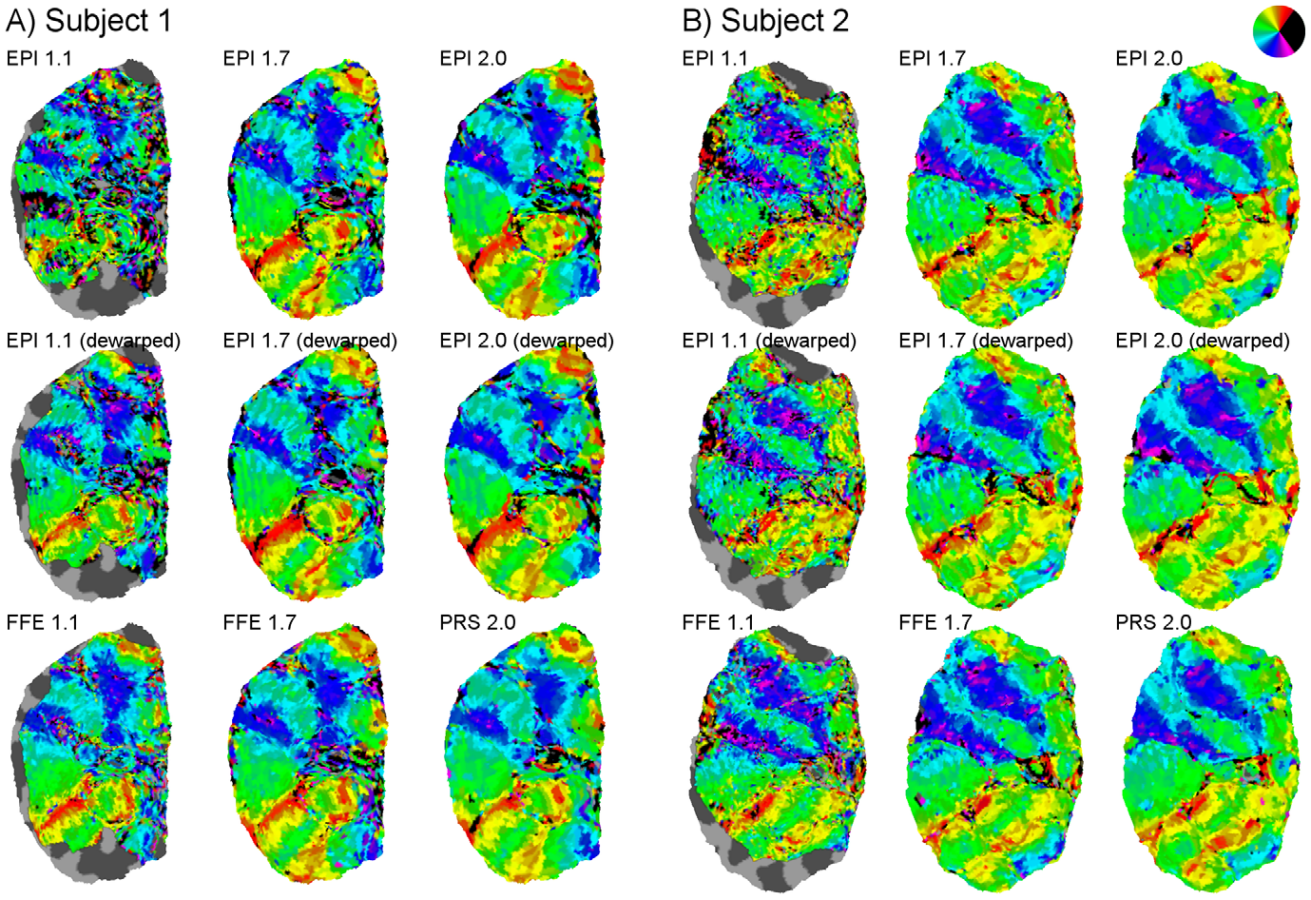
Retinotopic activation maps displayed on flattened patches of occipital cortex for the right hemispheres of two subjects. Response phase is shown in color for all voxels within the functional field of view, without statistical thresholding applied. Colored phases represent responses to positions predominantly in the contralateral visual field; phases corresponding to positions within the ipsilateral field are rendered in black (see inset). These apparent ipsilateral representations generally correspond to regions of low tSNR. Each map was obtained after less than ten minutes of scanning (see methods).

For visualization, the retinotopic maps were plotted on inflated and flattened [26], [34], [35] representations of the cortical surface of the occipital lobes (Fig. 3). Both sequence types produced consistent surface-based retinotopic maps of the early visual areas (Fig. 4; maps for all subjects and sequence types are available online as Information S1). Bands of activation corresponding to the borders between areas V1, V2, V3, V3A/B and hV4 [36] were clearly resolvable in a majority of hemispheres for both sequence types at all resolutions, and were in general highly reproducible across acquisition types. The maps appeared somewhat noisier at higher resolutions, and showed occasional signal dropouts or implausible (e.g. ipsilateral) response phase estimates, which were rendered in black. Inconsistent phase estimates near the foveal representation most likely reflect participants’ limited fixational stability, although this can be overcome through sufficient signal averaging and the use of highly practiced observers [37]. Further from the foveal confluence, such disruptions may be attributable to other sources, such as signal dropouts from nearby draining veins [38], or misalignment of the functional and structural images.

We examined a number of quantitative measures of the fMR signal across voxels identified as falling within the grey matter of area V1. These measures included temporal signal to noise, percent signal change, and the contrast to noise ratio. V1 was identified on the cortical surface, defined in the space of the anatomical images, by an automated parcellation scheme based solely on cortical folding patterns [39], [40]. This anatomical definition of V1 was chosen instead of one based on the retinotopic maps themselves so as to guarantee an independent and unbiased selection of the region of interest (ROI). Within this region, the aligned functional voxels were sampled at a depth of 75% of the distance from the grey matter/white matter border to the pial surface (measures at differing depths are described in more detail below). These measures were expected to reflect not only intrinsic properties of the sequences themselves, but also aspects of the quality of the functional to anatomical registration, as misalignments could result in functional activity shifting outside of the anatomically-defined V1 ROI.

**Figure 5:**
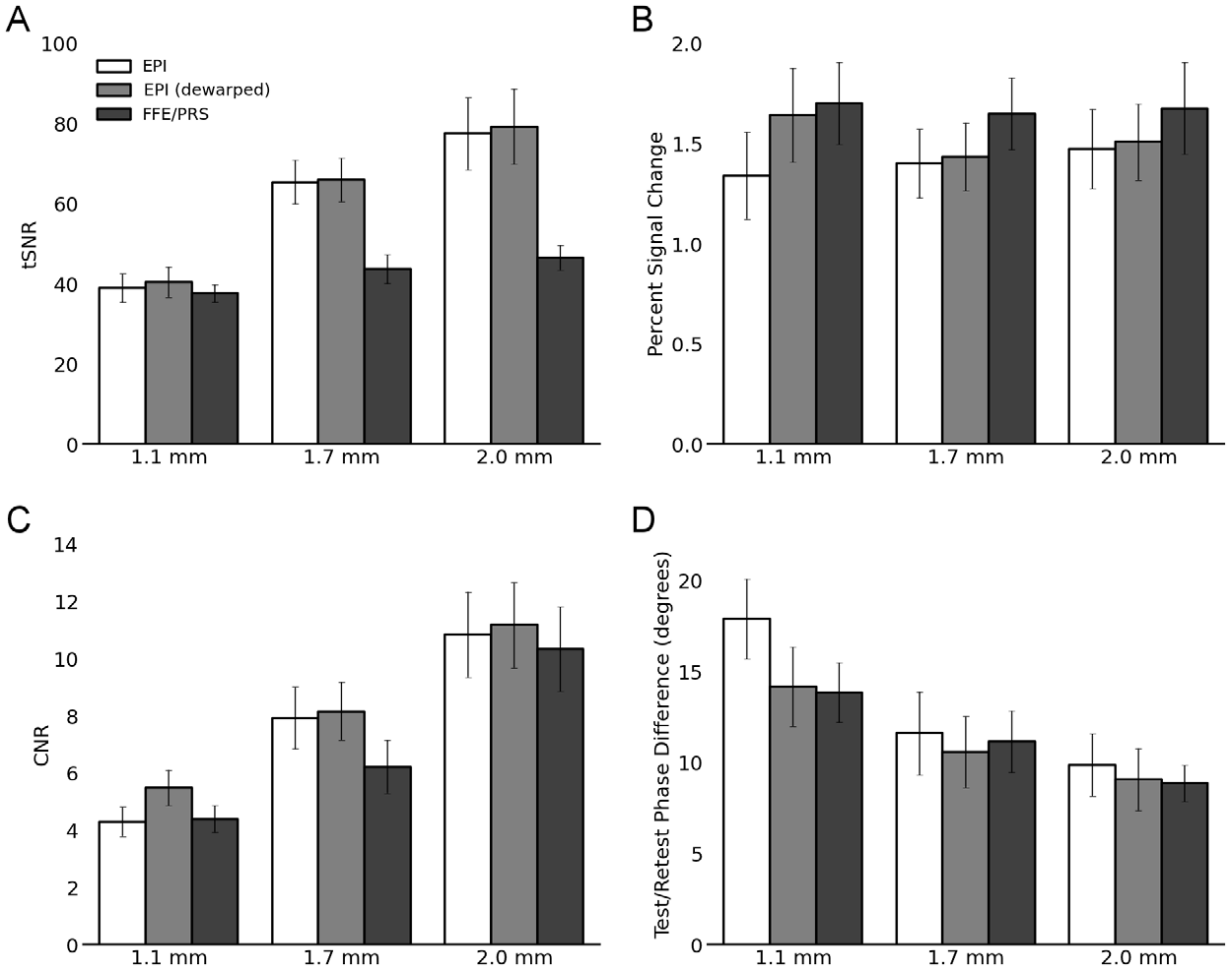
Quantitative measures by sequence type and resolution within V1 grey matter. Error bars indicate standard error of the mean across subjects. Variance within subjects was in general substantially smaller than that across subjects.

The temporal signal-to-noise ratio (tSNR) is often used as a measure of a pulse sequence’s sensitivity to functional activation (e.g. [41]). It was expected to increase at lower spatial resolutions, reflecting the greater signal available within larger voxels, and to be somewhat greater for single-shot EPI than multishot FFE. The median tSNR within V1 grey matter did indeed increase at lower spatial resolutions, and was substantially greater for the single-shot sequences at the 1.7 and 2.0 mm resolutions (Fig. 5A). Within-subjects analysis of variance showed that the effects of sequence type (FFE, EPI, or EPI without dewarping) reached significance only at the 1.7 and 2.0 mm resolutions (each *p*<10^-3^). Though the tSNR within the identified grey matter voxels was not greatly increased by field map based dewarping, it was consistently slightly higher than for single-shot EPI without dewarping. This was a small effect relative to the difference in measured tSNR across subjects, but when this variability across subjects was accounted for in a paired t-test of EPI tSNR with and without dewarping, dewarping led to a significant (*p*<0.05) improvement in tSNR at the 2.0 mm resolution.

Since the EPI and FFE sequences were segmented differently in the *ky* plane, the resulting images could be expected to show differing patterns of Nyquist ghosts, which might have a strong impact on tSNR measurements if ghosts were to impinge on the V1 ROI in one sequence type but not another. However, no strong Nyquist ghosting was evident in any set of images, and temporal noise and tSNR maps did not show any systematic spatial structure consistent with the presence of substantial ghosts (Information S2). It is therefore unlikely that differential Nyquist ghosting contributed strongly to the differences in measured tSNR between sequences.

Signal change was comparable across resolutions, though slightly greater for the multishot sequences (Fig. 5B). This effect of sequence type was statistically significant, as indicated by within-subjects ANOVAs performed at each resolution (all *p*<0.05). FFE sequences consistently resulted in greater signal change than EPI without dewarping (all paired t tests at *p*<0.05 across resolutions). FFE led to significantly greater signal change than EPI with dewarping at 1.7 mm (*p*<0.01), and showed a trend in this direction at 2.0 mm (*p*<0.1). This difference in signal change between the FFE and dewarped EPI sequences was unexpected, but may be due to a difference in the balance of T1 versus T2* weighting between the sequence types, stemming from the much shorter TR of the FFE sequences. Alternately, it may simply reflect inaccuracies in the alignment of the EPI images with the V1 ROI that persist even after distortion correction. Signal change for EPI with and without distortion correction trended towards a significant difference only at 1.1 mm resolution (*p*<0.1).

The contrast-to-noise ratio (CNR) is a direct measure of functional sensitivity. It is closely tied to measures commonly used to evaluate the statistical significance of functional activation, but is independent of the number of time points sampled (since the number of time points acquired varied across sequences, statistical significance was not directly evaluated here).

Similarly to the tSNR measurements, CNR was expected to increase at lower resolutions, and to be somewhat higher for EPI than for FFE. This was indeed found to be the case (Fig. 5C). Notably, although the volume of a single voxel dropped nearly sixfold between the low- and high-resolution sequences, CNR decreased by only a factor of roughly 2.5. Within-subject ANOVAs showed significant (both 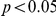) effects of sequence type on CNR at the 1.1 and 1.7 mm resolutions, and a trend towards a significant effect (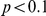) at 2.0 mm resolution. The dewarped EPI images showed significantly higher CNR than the FFE sequences at 1.1 and 1.7 mm resolutions (both 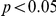), and a trend towards significantly greater CNR at 2.0 mm (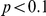). Without dewarping, EPI showed significantly greater CNR than FFE only at the 1.7 mm resolution (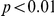). The difference in CNR between EPI with and without dewarping was itself significant only at the 2.0 mm resolution (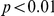).

We also assessed the precision and reliability of the retinotopic maps through a test/retest comparison of the estimated response phase at each voxel. Each of two functional runs acquired at each combination of sequence type and resolution were processed separately, giving rise to two independent estimates of the response phase at every point. A phase error measure was defined as the median absolute difference in phase estimates across the grey matter voxels of V1. This phase error metric improved for larger voxel sizes, and was comparable for the multishot and dewarped single-shot EPI sequences across all resolutions (Fig. 5D). Within-subject ANOVAs showed that sequence type significantly affected the reliability of the retinotopic maps only at the highest (1.1 mm) resolution (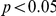). This effect was driven solely by the EPI data without dewarping, which showed significantly lower reliability than either FFE or EPI with dewarping (both 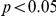).

**Figure 6:**
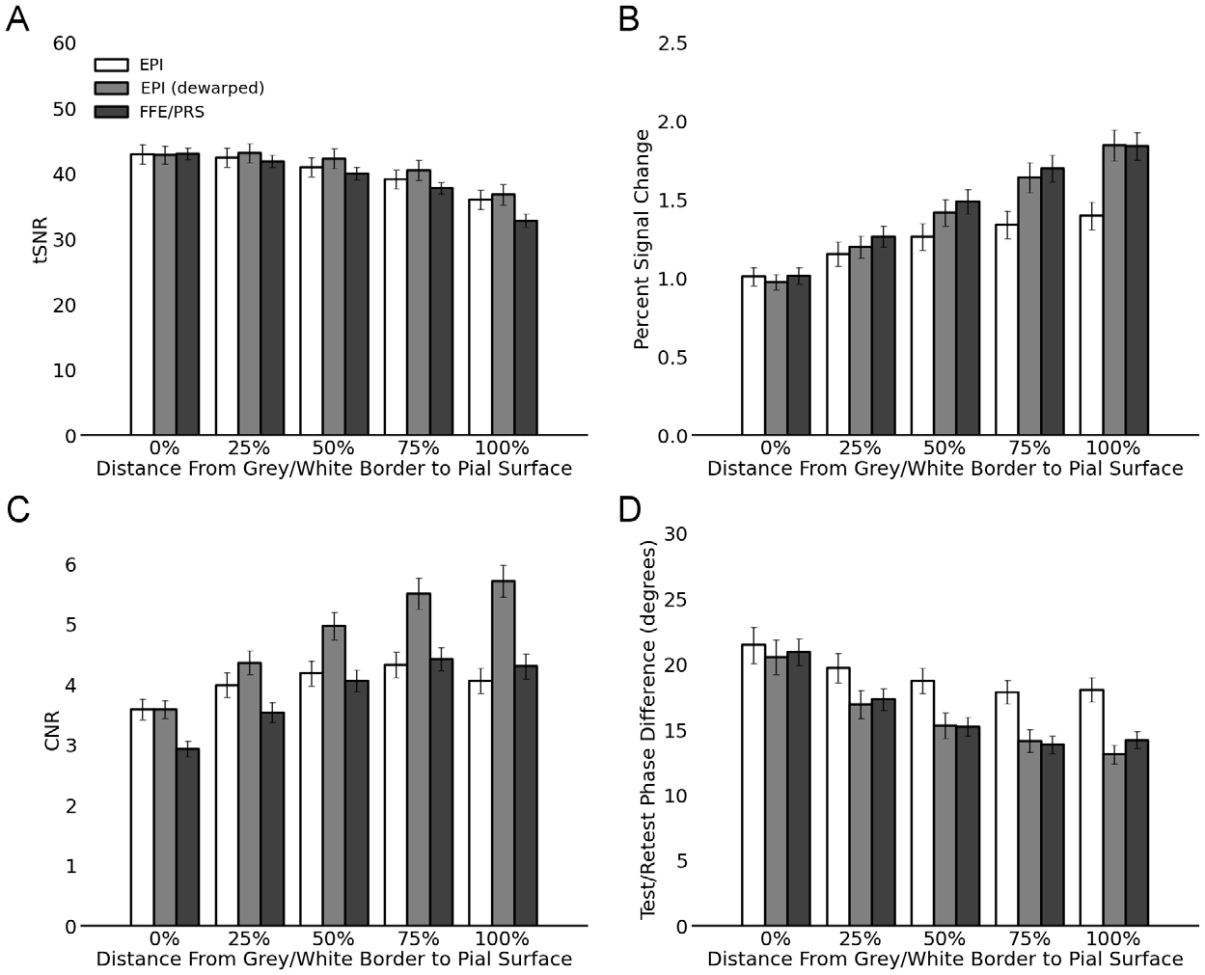
Quantitative measures by sequence type and cortical depth within V1 grey matter. Data shown are from the 1.1 mm resolution sequences. Lower resolutions showed trends that were similar but smaller in magnitude. Error bars indicate s.e.m. across subjects.

When voxel size becomes small relative to the thickness of cortical grey matter, the results of surface-based analyses may be heavily influenced by the depth at which the functional voxels are sampled [20], [42], [43]. Larger veins run along the pial surface of cortex, with smaller vessels descending into the depths of the grey matter [44]. Sampling high-resolution fMRI data at differing depths within the grey matter thus may be expected to change the relative contribution of the pial veins versus the intracortical vasculature, with potentially substantial effects on the resulting surface-based maps [20]. We resampled the fMRI retinotopic activation patterns at several different depths (0, 25, 50, 75, and 100% of the distance from the grey matter/white matter border to the pial surface), and measured the median tSNR, percent signal change, CNR, and test/retest reliability of the phase estimate at each depth within the V1 grey matter. In agreement with previous reports using gradient-echo fMRI [20], [43], in our high-resolution (1.1 mm) data we found that the median percent signal change increased steadily approaching the pial surface. Although tSNR declined in the superficial levels of the grey matter, both CNR and test/retest reliability reached their maximum at or slightly below the pial surface (Fig. 6). Interestingly, although the measures of the EPI sequence with and without dewarping diverged near the cortical surface, at lower depths dewarping had little effect, suggesting that the benefits of dewarping stem primarily from improvements in alignment around the grey matter/CSF border. At lower resolutions, changes in position within the cortical grey matter become less substantial relative to the effective size of a voxel. Although the 1.7 and 2.0 mm resolution data showed similar trends as the 1.1 mm resolution data, the effects of sampling depth were accordingly less pronounced (data not shown).

Different stimulus wedge angles were used across participants, which might be expected to contribute to the variability between subjects in signal change and CNR. Importantly, each individual subject always viewed a wedge stimulus of the same width across sequences and resolutions, preserving the validity of the within-subject ANOVAs used above. The temporal pattern of stimulation produced at any given point on the retina by a rotating wedge can be considered as a periodic pulse train, with duty cycle determined by the wedge angle. For example, a 180° hemifield wedge would result in temporal stimulation with a 50% duty cycle, a 90° quarter-field wedge would lead to a 25% duty cycle, and so on. Assuming that the response of visual cortex can be well approximated as a linear system, the resulting cortical time series would be expected to share a similar duty cycle. (In practice, the larger receptive fields found in higher visual areas would likely lead to a somewhat longer cortical duty cycle than that of the stimulus itself.) The amplitude at the fundamental frequency of a periodic pulse train with duty cycle *d* is proportional to *dsinc*(*d*), which is nearly linear so long as *d* is substantially less than 0.5. We therefore expect that increasing wedge angle should lead to an approximately linear increase in signal change and CNR in V1, up to wedge angles approaching 180°. After averaging across sequence types and resolutions, we find a trend in the expected direction for signal change (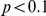), suggesting that these differences in wedge angle may indeed account for some of the between-subjects variability in these measures.

## Discussion

Geometric distortion is widely acknowledged to be a concern in functional MRI, particularly as field strengths and spatial resolutions increase. However, the practical impact of uncorrected distortion on subsequent analyses and results has been examined only infrequently [11], [12]. In the absence of such evidence, it is difficult to evaluate the utility of schemes for correcting or controlling distortion. The benefits to be obtained by effectively managing distortion may be unclear, and will likely depend strongly on the precise experimental question being addressed.

We find that geometric distortion has appreciable qualitative and quantitative impacts on retinotopic mapping at a resolution of 1.1 mm. Although high-resolution, parallel-accelerated single-shot EPI led to recognizable retinotopic maps even without distortion correction, in many cases the maps were notably improved by field map based dewarping. Dewarping of the 1.1 mm EPI images increased CNR within voxels identified as V1 grey matter in the anatomical images, and also improved the test/retest reliability of the retinotopic maps. These improvements can be attributed to better alignment of the functional and anatomical images after dewarping, as more true functional activity comes to fall within the anatomically-defined V1 ROI. At lower resolutions the impact of distortion correction was less pronounced, although small improvements in tSNR and CNR were still observed. The multishot sequences generally showed somewhat lower CNR than their single-shot counterparts. However, the retinotopic maps produced from these sequences were qualitatively quite comparable to those from dewarped single-shot EPI, and demonstrated similar test/retest reliability, with no additional distortion correction required. Such multishot sequences may thus represent a plausible alternative to single-shot EPI at high field strength, particularly when surface-based analyses of high-resolution fMRI data are planned.

Two previous studies have also examined conventional retinotopic mapping at high field strength. Hoffmann and colleagues [45] used accelerated single-shot EPI sequences at both 3T and 7T at isotropic resolutions of 2.5, 1.4, and 1.1 mm, finding greater sensitivity and a larger extent of functional activation at 7T. Olman and coworkers [46] compared gradient-echo and spin-echo single-shot EPI sequences at 7T at an isotropic resolution of 1.8 mm, finding comparable maps but a higher CNR for the gradient-echo sequences. These authors also noted the necessity of distortion correction to achieve good alignment of the functional and anatomical images for surface based reconstruction.

More recently, Polimeni and colleagues [20] exploited the regular retinotopic organization of V1 in an innovative investigation of the laminar spread of the BOLD signal at 7T. Using a model of the human cortical magnification function, these authors designed a visual stimulus to evoke cortical activation in the shape of the letter ‘M’. The measured activation pattern, detected by accelerated single-shot EPI at 1 mm resolution, agreed most closely with the predicted pattern in deeper layers of the grey matter. Here we find that the reproducibility of the retinotopic maps is greatest in more superficial layers, likely reflecting the larger BOLD signal and higher CNR seen closer to the cortical surface. Greater signal change near the superficial layers is commonly found in gradient-echo fMRI (e.g. [20], [43]), and is generally attributed to the proximity of larger draining veins along the cortical surface, which may result in displacement of the fMR signal [47]. The selection of an appropriate cortical depth for high-resolution fMRI analyses may therefore best be viewed as a tradeoff between greater CNR and precision (reproducibility) closer to the cortical surface, and greater fidelity to the true underlying patterns of neural activity near the grey matter/white matter boundary.

Single-shot EPI has come to dominate other fMRI data acquisition techniques. However, much of the conventional wisdom supporting the use of single-shot EPI developed out of the early years of fMRI research, at lower resolutions and field strengths, where the advantages of multishot techniques in terms of achievable resolution and insensitivity to distortion were generally not found to justify the loss of sensitivity to functional activation [48]. The growing use of very high field scanners for high-resolution fMRI may justify revisiting some of these issues. A recent study [49] comparing single-shot EPI to 3D-FFE and PRESTO sequences using the NPAIRS framework [50] found that although high-resolution EPI offered greater functional sensitivity, in terms of statistical z-scores and better prediction within the NPAIRS scheme, FFE led to similar reproducibility and substantially improved geometric distortion.

We find that both accelerated single-shot EPI and accelerated multishot FFE sequences represent viable options for high-resolution fMRI at 7T. Both sequence types led to qualitatively comparable retinotopic maps, with equivalent test/retest reliability. However, EPI sequences were found to offer generally higher tSNR and CNR than their FFE counterparts after field map based distortion correction was applied. Distortion correction substantially improved the surface-based EPI maps, particularly at the highest resolution tested (1.1 mm), where without distortion correction the EPI results were no better than their FFE equivalents. It is likely that these trends would continue at still higher resolutions, making multishot pulse sequences increasingly appealing alternatives to single-shot EPI at spatial resolutions of 1 mm and below.

## Methods

### Ethics Statement

This study was approved by the Institutional Review Board of Vanderbilt University. Signed informed consent was obtained from all participants.

### Subjects

Eight subjects (three male) participated in this study. All had normal or corrected-to-normal vision, and had participated in previous fMRI studies. Data from 6 of these 8 subjects was fully analyzed. Technical difficulties prevented a full data set from being acquired in one individual, while the remaining subject, who had not previously participated in visual fMRI studies, showed very poor maps in which the early visual areas could not be clearly identified. This was presumably due to poor fixation, though as eyetracking was not available in the 7T scanner this could not be independently verified.

### Experimental Stimuli

The subjects observed counterphasing (8 Hz), clockwise rotating black and white checkerboard wedges, which were projected (Avotec, Stuart FL) onto a screen mounted in the scanner bore above the chest, and viewed through a mirror attached to the MR head coil. Differing subjects viewed wedges of differing angles, with 108° wedges used for 3 of the analyzed participants, 72° for 2 participants, and a 45° wedge for the remaining analyzed subject. The wedges rotated about fixation at a speed of 1 cycle every 36s. At random intervals (once every 6.67s on average), a randomly positioned wedge segment dimmed for 0.5s. Participants were instructed to respond each dimming event with a button press, while maintaining fixation at the center of the screen. The dimming events were relatively easy to detect, with subjects responding within 1 second on 89% of trials (minimum across subjects 78%, maximum 97%).

### Magnetic Resonance Imaging

MR scanning was performed on a 7T Philips Achieva magnet located at the Vanderbilt University Institute of Imaging Science, using a 16 channel receive-only head coil with an outer quadrature transmit coil (Nova Medical, Wilmington MA). A bite bar was used to minimize subject head motion. Functional imaging was performed at each of 3 isotropic resolutions (1.1, 1.7 and 2.0 mm) using both single-shot 2D-EPI and multishot 3D fast field echo (FFE) sequences. Like single-shot EPI, FFE pulse sequences use gradient echos with Cartesian echo train readouts. Here, the 3D-FFE readouts were segmented so that initially, a single *ky* plane was acquired across 3 RF shots in a standard interleaved segmentation pattern. After the *ky* readout was finished, *kz* was incremented and the next *ky* plane acquired as above, until the entirety of the readout volume was completed.

The total duration of a single functional run was chosen to be 288 s, corresponding to 8 stimulus cycles at 36 s/cycle. Two runs were acquired for each subject and each combination of resolution and sequence type. B0 field map images were also acquired with resolution and field of view matching that of each functional sequence.

Imaging parameters (Table 1) were chosen first to achieve the desired field of view and voxel dimensions (or equivalently matrix size). Echo time was chosen to be 22 ms for most sequences, near previously reported values for T2* of cortical grey matter at 7T [51]. RF spoiling was used in all multishot sequences.

Parallel SENSE acceleration was applied in the phase encode (right/left) direction. The degree of parallel acceleration (*R*) for each sequence was chosen by considering the geometry factor (*g*), which measures the degree to which intrinsic thermal noise sources are amplified by the SENSE image reconstruction process. [15]. *g* varies spatially, as determined by the coil sensitivity profiles and degree of correlated noise across coils, and increases with greater levels of parallel acceleration. For each sequence we acquired maps of the *g*-factor within the field of view and chose acceleration levels so that *g* did not exceed 2 at any point in the image. This resulted in acceleration (undersampling) levels ranging between 2.2 and 2.7 (Table 1). While in 3D sequences parallel acceleration can be applied simultaneously in both phase encode directions, here the relatively narrow depth of the imaging slab in the anterior-posterior dimension prevented effective acceleration in this direction without incurring a substantial cost in *g*.

The repetition time (TR) of the single-shot sequences was initially chosen to be as short as possible, given the constraints of matrix size, number of slices, echo time, and degree of parallel acceleration, and was then lengthened slightly so as to ensure a total acquisition time that evenly divided the desired 288s run length. For the multishot sequences, after fixing the above constraints, the echo train length was chosen to be roughly a factor of 3 shorter than that of the equivalent single-shot sequence. TR was then set to be the shortest value that resulted in an acquisition time which divided 288s, as with the single-shot sequences. Flip angles were set at or near the Ernst angle for each TR.

For the multishot sequence at 2.0 mm resolution, after fixing field of view, resolution, echo train length, and level of parallel acceleration, substantial “dead time” remained in the acquisition between the RF pulse and the beginning of the readout. By slightly increasing the effective echo time to 26 ms, this dead time gap could be made longer than the total readout duration. This allowed an additional echo shifting gradient to be applied [30], converting the sequence into 3D-PRESTO (PRinciple of Echo-Shifting with a Train of Observations) [31], [32]. In these sequences, echo formation is delayed until after a second RF excitation pulse is emitted. The echo resulting from the first pulse then forms within what would otherwise be dead time between the second excitation and its associated readout. By interleaving pairs of RF pulses and readouts in this manner total scan time can be considerably reduced. The signal intensity in RF-spoiled PRESTO sequences is slightly reduced by interference from the intervening RF pulse between excitation and readout [52], [53], but this may have a limited effect on tSNR in low-resolution regimes where physiological noise sources dominate thermal noise.

The anatomical images used for cortical reconstruction were acquired during separate imaging sessions on a 3T Philips Achieva MR scanner using an 8-channel head coil. Anatomical imaging used a T1-weighted turbo field echo (TFE) sequence at 1 mm isotropic resolution (TR 8 ms, TE 3.7 ms).

### Data Analysis

Each subject’s T1-weighted anatomical images, acquired at 3T, were processed using Freesurfer [34], [35]. The Freesurfer reconstruction produces polygonal meshes corresponding to the grey matter/white matter border and the pial surface of cortex for each hemisphere. Between these two surfaces, three additional intermediate grey matter surfaces were created at 25%, 50%, and 75% of the depth of the grey matter using the Freesurfer program *mris_expand* [20].

The 7T functional data were first motion-corrected using FSL’s mcflirt [54] with 6 degrees of freedom and sinc kernel interpolation. Within-run motion never exceeded 2 mm, and averaged less than 0.5 mm. Runs with the same pulse sequence and resolution were aligned to the same target volume, defined as the mean of the first run of the set to be acquired.

FSL’s *fugue* software was used for distortion correction of the single-shot EPI data [8]. As the interpolation inherent to dewarping results in some degree of spatial smoothing, in order to maintain a fair comparison between sequence types distortion correction was applied only to the volumes of statistical results and (for alignment purposes) the mean functional images, rather than to the full time series data. B0 field map images were acquired using gradient-echo sequences with fields of view matching that of the functional data for each resolution. To correct for any slight head movements that may have occurred between the field map acquisition and that of the functional data, the magnitude component of the B0 field map was first warped to match the expected distortion of the functional data, and then aligned to the corresponding mean motion-corrected functional image using FSL’s *flirt*, with 6 degrees of freedom and a correlation ratio cost function. After verifying the accuracy of the registration, the resulting transform matrix was then applied to the field map itself, bringing it into alignment with the motion-corrected functional data. The field map image was expected to be relatively free of distortion, as it was acquired using a gradient-echo sequence without EPI readout. It was therefore not itself dewarped. The field map was smoothed by a 16 mm FWHM Gaussian kernel prior to dewarping of the EPI images.

After motion correction and (where applicable) dewarping, the mean functional image at each sequence and resolution was aligned to the anatomical image used for the cortical reconstruction. First an approximate manual alignment was performed using Freesurfer’s *tkregister2* program, then this manual registration was used as a starting point for an automated boundary-based alignment using the Freesurfer program *bbregister* [33]. This algorithm attempts to position the functional volume so as to maximize the change in pixel intensity across the grey matter/white matter boundary defined from the anatomical images. A previous study found that a similar alignment procedure was sufficiently accurate as to resolve changes in activation across the depth of cortex using high-resolution (1 mm) 7T single-shot EPI data [20]. In 3 cases, all involving multishot data, the automated alignment required final manual correction. These failures of the automated registration may be due to reduced grey/white contrast in the multishot images relative to single-shot EPI, stemming from the much shorter TR of the multishot acquisitions. Alignment, including the initial manual stage, was performed separately for the single-shot EPI data with and without distortion correction applied.

Regions of interest (ROIs) for V1 in each hemisphere were defined on the reconstructed cortical surface. To avoid any possible bias that might result from using the same functional data first to define an ROI and then measure activity within the same region, the V1 ROIs were defined by an automated procedure based purely on anatomical cortical folding patterns [39], [40]. These surface-based ROIs were then restricted to include only points within the field of view (after motion correction) of both the single and multishot 1.1 mm functional images. The field of view of these high-resolution acquisitions was contained within the larger fields of view of the lower resolution scans.

For the retinotopy analysis, linear trends were first removed from the time series of each functional volume, then the detrended time series were averaged together. The amplitude and phase of the Fourier component at the 8 cycles/run stimulus frequency was then calculated by a fast Fourier transform. Under the null hypothesis of no activation and temporally white Gaussian noise, the Fourier power at the stimulus frequency is distributed as a *x* ^2^ random variable with 2 degrees of freedom. The average estimated power across all other “noise” frequencies is then distributed proportionately to a *x* ^2^ random variable with 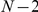 degrees of freedom, where *N* is the number of points in the functional time series. The ratio of the power at the stimulus frequency to the average noise power is then distributed as an F statistic with 2 and *N*–2 degrees of freedom under the white noise null hypothesis. In calculating the estimated noise power for fMRI time series, the white noise assumption was relaxed slightly by excluding from the estimate those Fourier components corresponding to the time series mean, low frequencies up to 2 cycles per run, the fundamental stimulus frequency and its first and second harmonics, and frequencies within 1 cycle/run of the fundamental or harmonics. Excluding these components reduced the degrees of freedom of the noise power estimate to *N*–24. For display, those voxels where the power at the stimulus frequency exceeded threshold (as determined by this F statistic) were rendered in color according to the phase of the complex Fourier component at the stimulus frequency.

Quantitative measures included tSNR, percent signal change, and CNR. For summary purposes, the median across identified V1 voxels was first calculated for each subject, and then statistical tests were performed on these median scores across subjects. tSNR was defined as the timecourse mean intensity divided by its standard deviation, after removing linear trends and periodic components corresponding to the stimulus frequency and its first two harmonics. The stimulus-related periodicities were removed in order to make these measurements more comparable with resting-state measurements, as are typically reported. Percent signal change was measured as the ratio of the amplitude of the timecourse Fourier component at the stimulus frequency to the timecourse mean. CNR was quantified as the square root of the F-statistic used to determine the significance of the functional activation (see above). It is thus roughly comparable to a z-score measure, as would be used to determine CNR for a typical scalar (non-phase-encoded) activation design. If the time series noise were temporally independent and normally distributed, this CNR measure would also be proportional to the precision (1/standard deviation) of the estimated response phase [55]–[57].

## Supporting Information

Information S1 **Retinotopic maps.** The early visual areas of both hemispheres are shown on flattened occipital patches for all sequences and participants.
(PDF)

Information S2 **Representative mean, temporal noise, and tSNR images.** Mean functional, noise standard deviation, and temporal signal to noise volume images are presented for the 1.12 mm EPI and FFE sequences in an example subject.
(PDF)
