# Supplementary material for: Multishot versus Single-Shot Pulse Sequences in Very High Field fMRI: A Comparison Using Retinotopic Mapping
Source: PLoS One. 2012 Apr 13;7(4):e34626. doi: 10.1371/journal.pone.0034626 (PMC3326057; doi:10.1371/journal.pone.0034626)
Supplement: Information S1 — Retinotopic maps. The early visual areas of both hemispheres are shown on flattened occipital patches for all sequences and participants. (PDF) [file pone.0034626.s001.pdf]

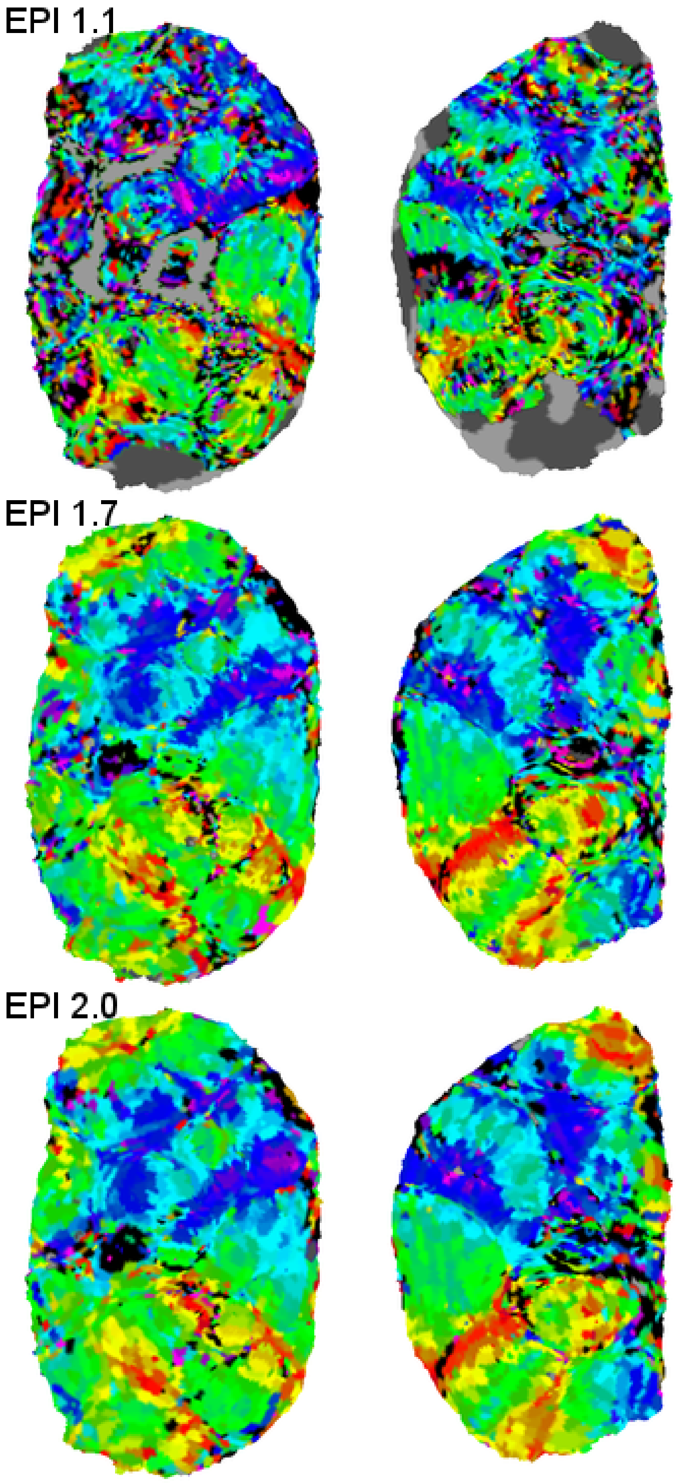

Supplementary Figure 1. Subject 1: EPI

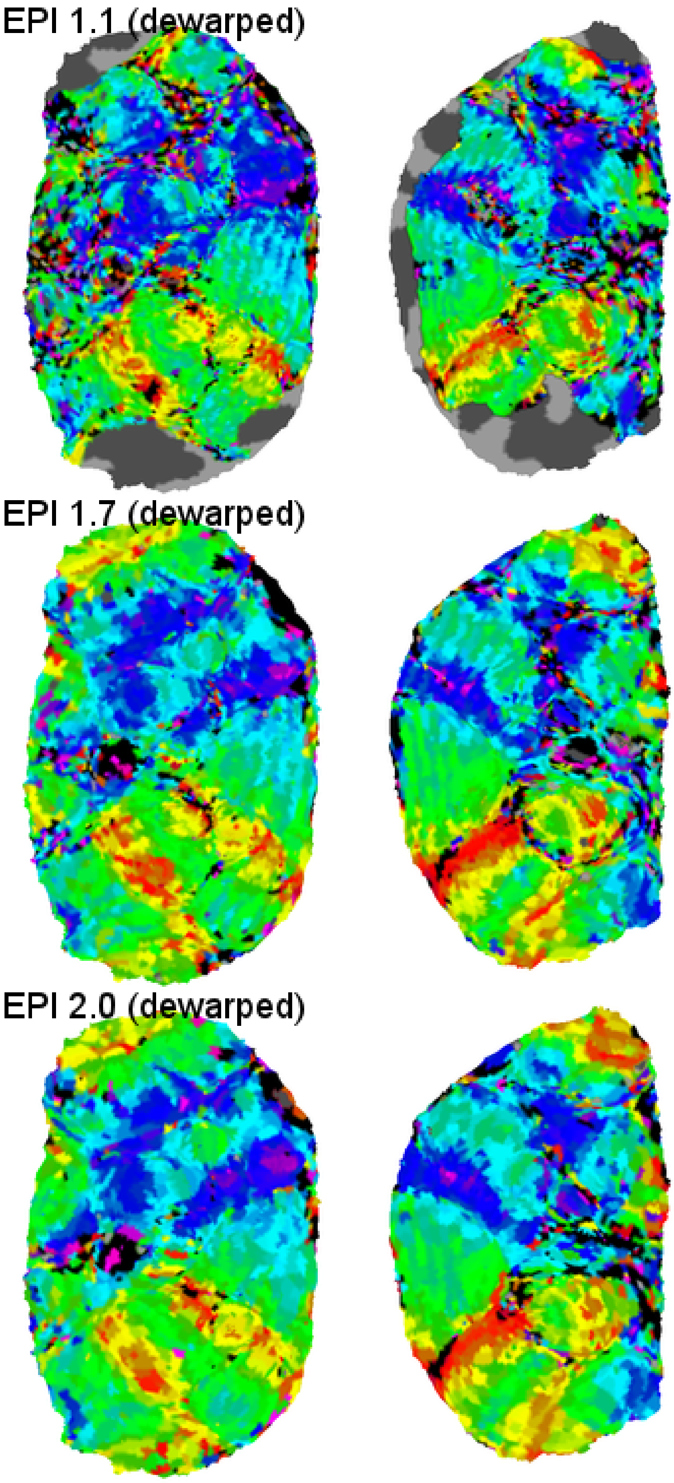

Supplementary Figure 2. Subject 1: EPI (dewarped)

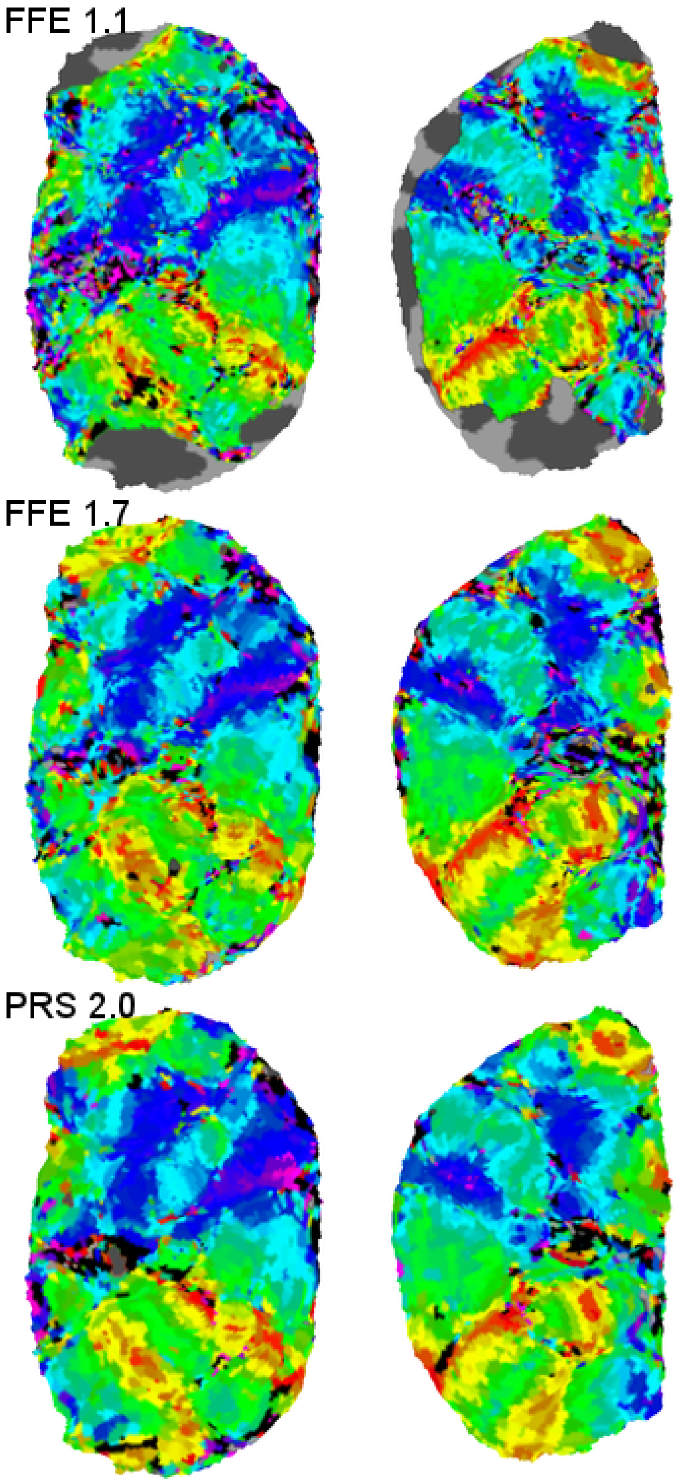

Supplementary Figure 3. Subject 1: FFE/PRESTO

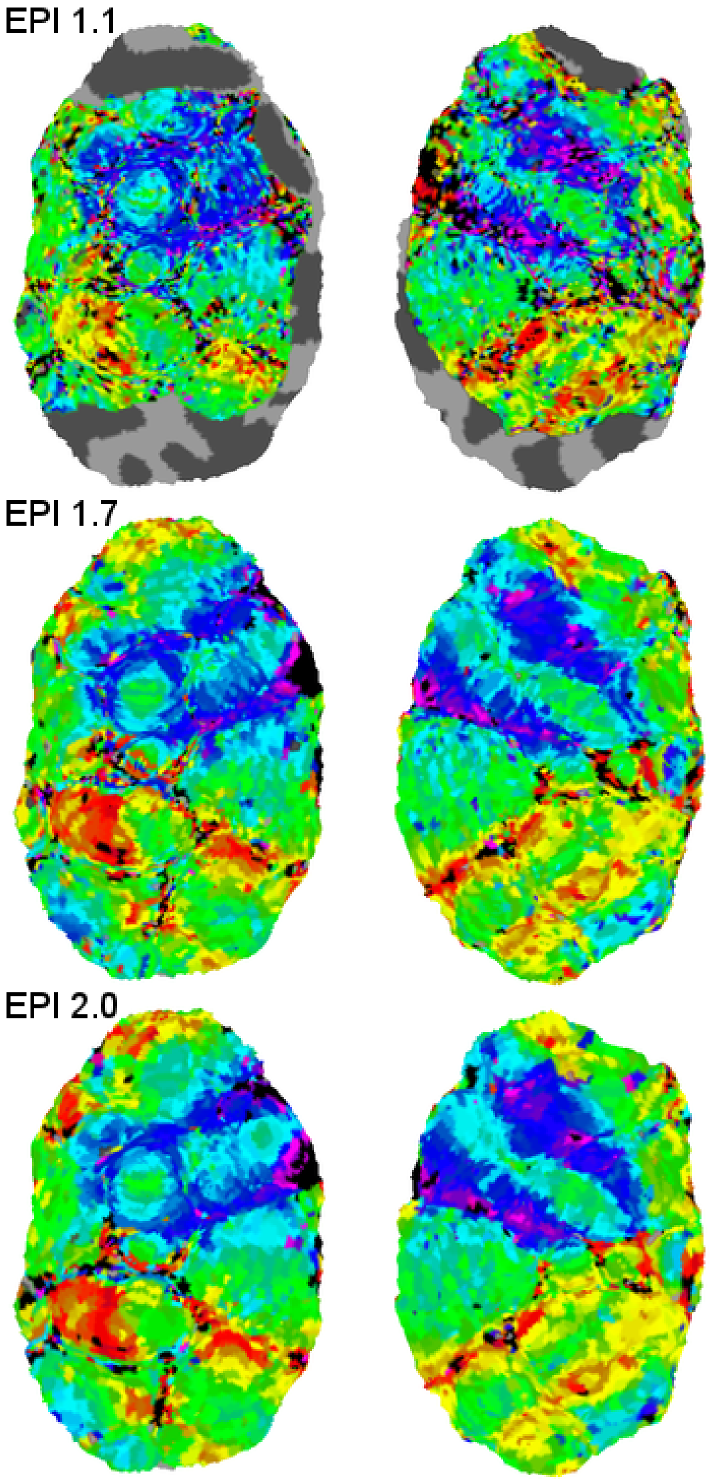

Supplementary Figure 4. Subject 2: EPI

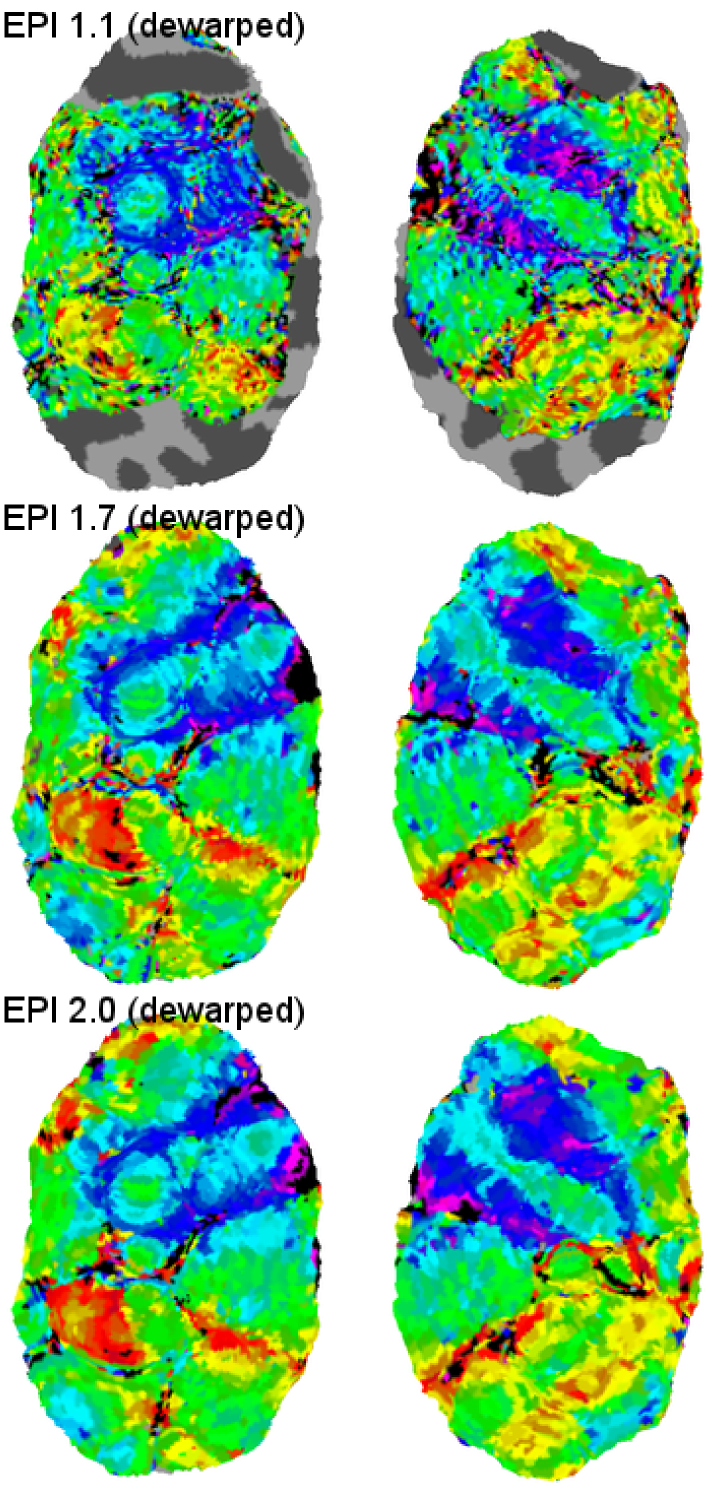

Supplementary Figure 5. Subject 2: EPI (dewarped)

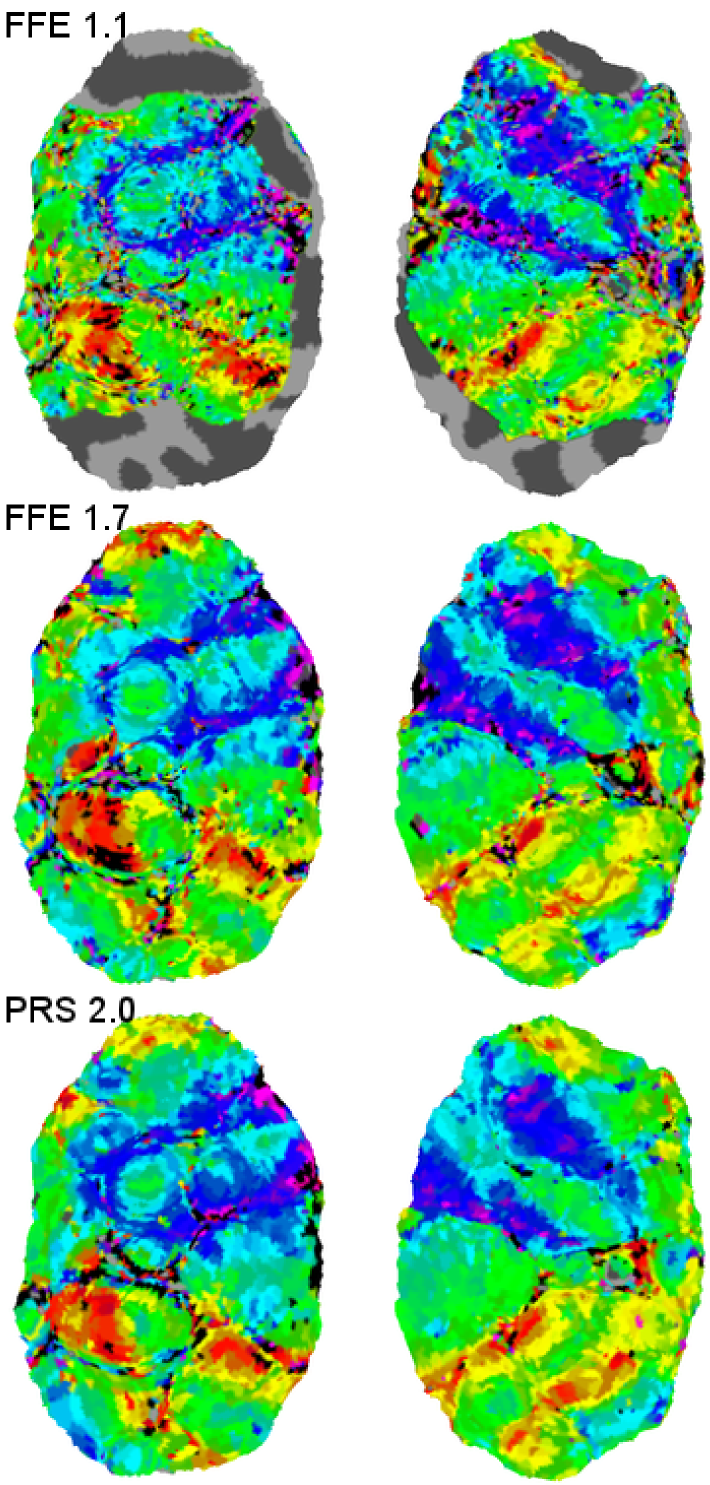

Supplementary Figure 6. Subject 2: FFE/PRESTO

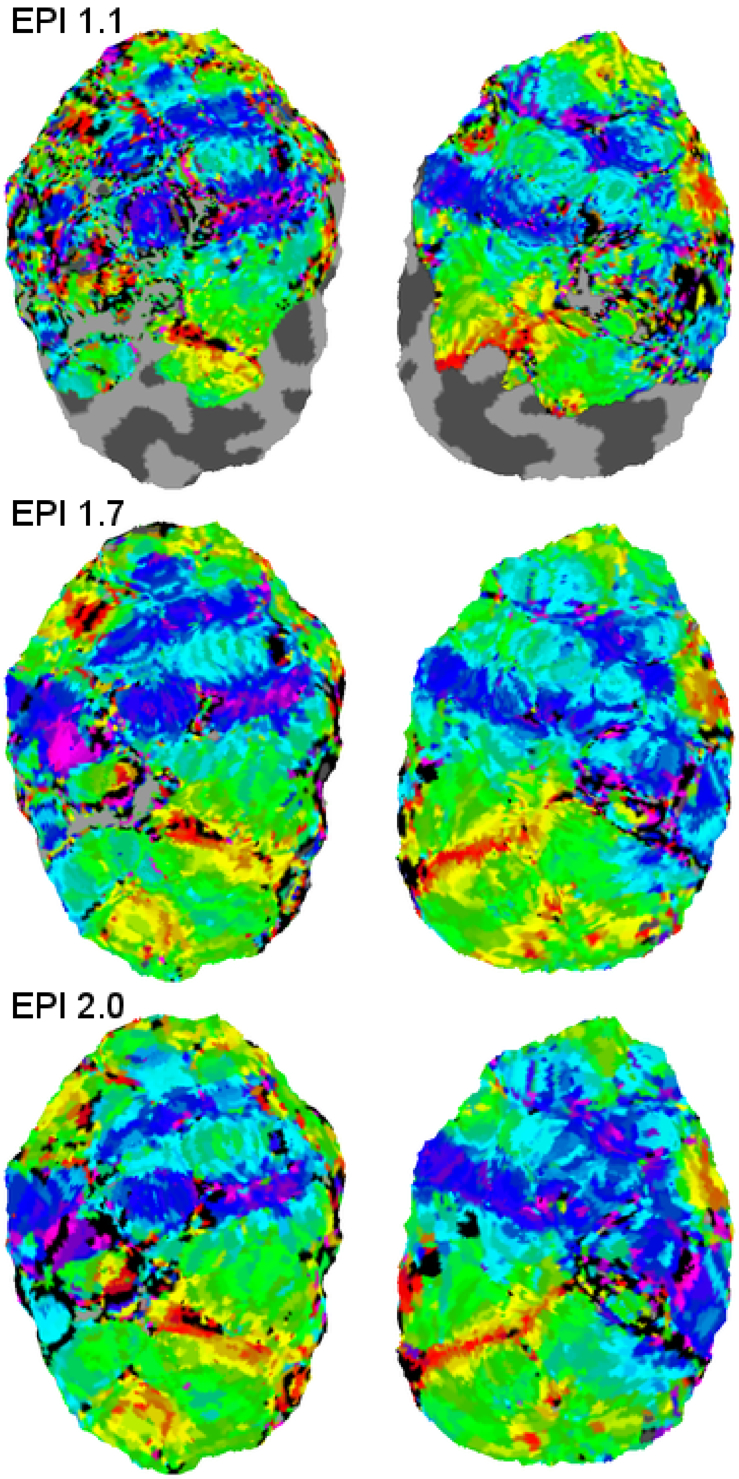

Supplementary Figure 7. Subject 3: EPI

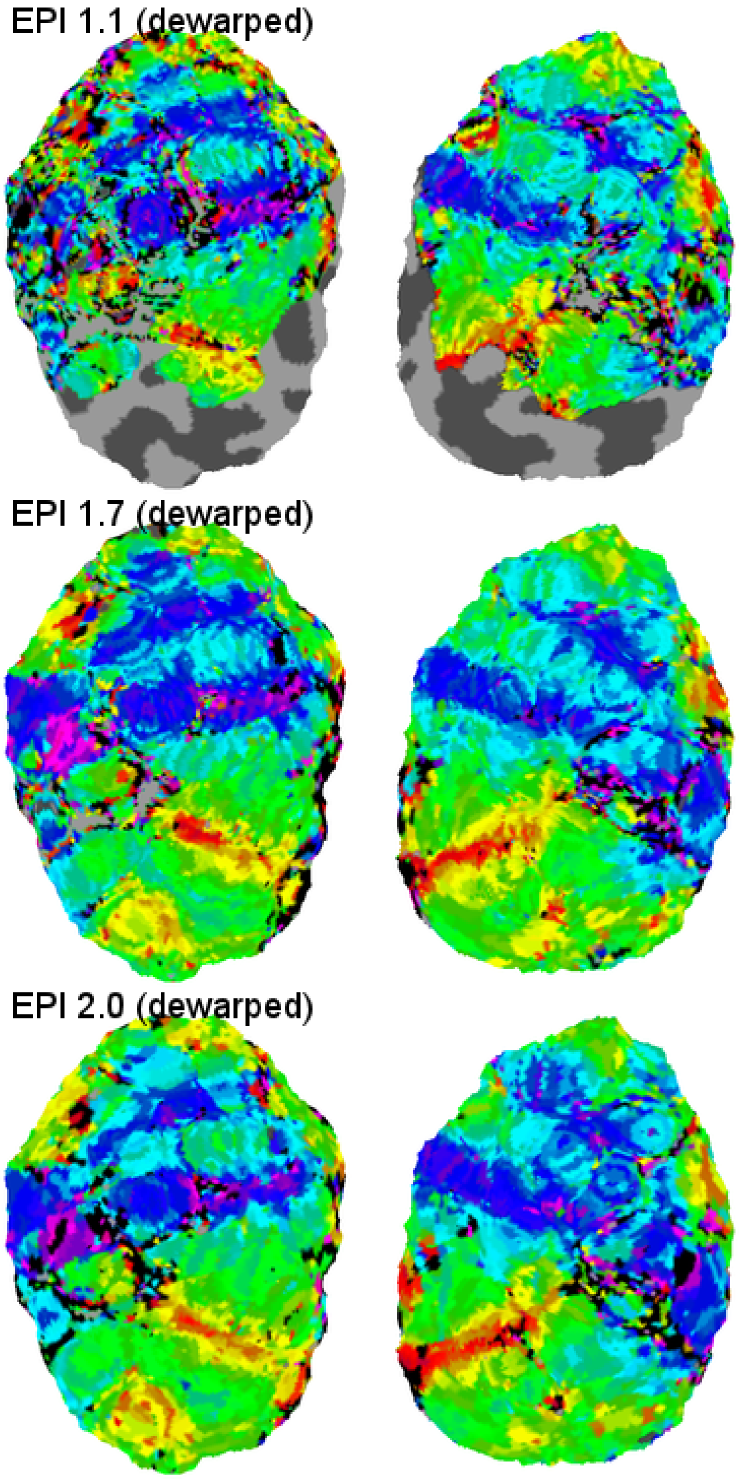

Supplementary Figure 8. Subject 3: EPI (dewarped)

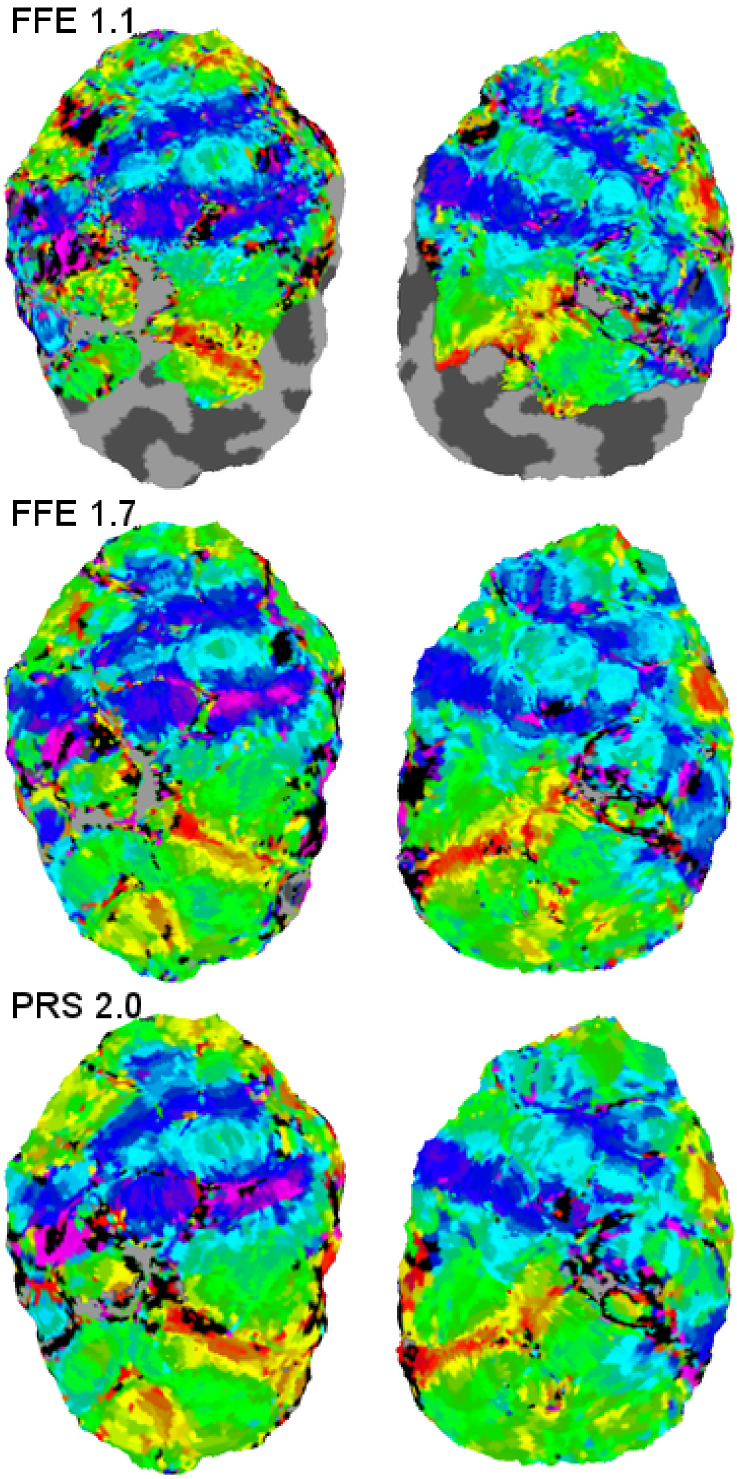

Supplementary Figure 9. Subject 3: FFE/PRESTO

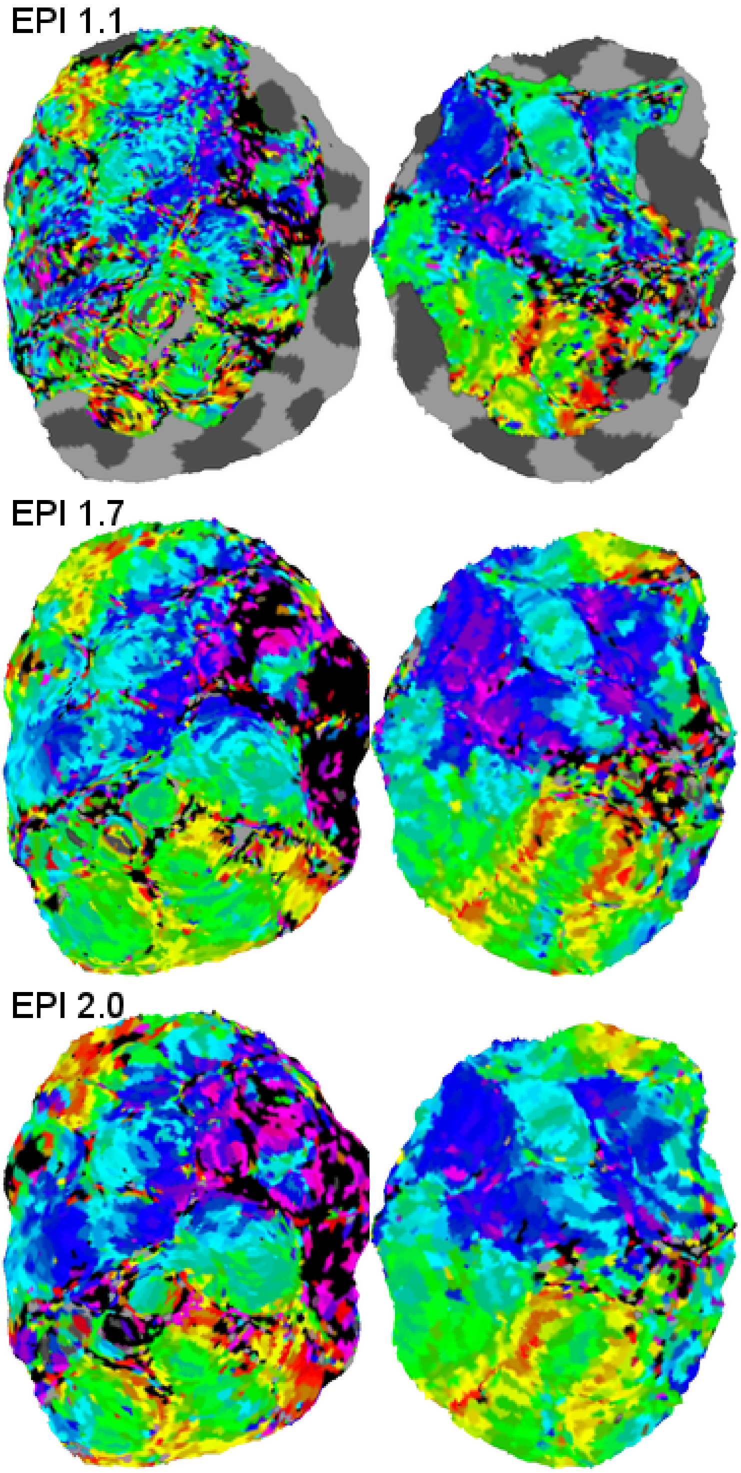

Supplementary Figure 10. Subject 4: EPI

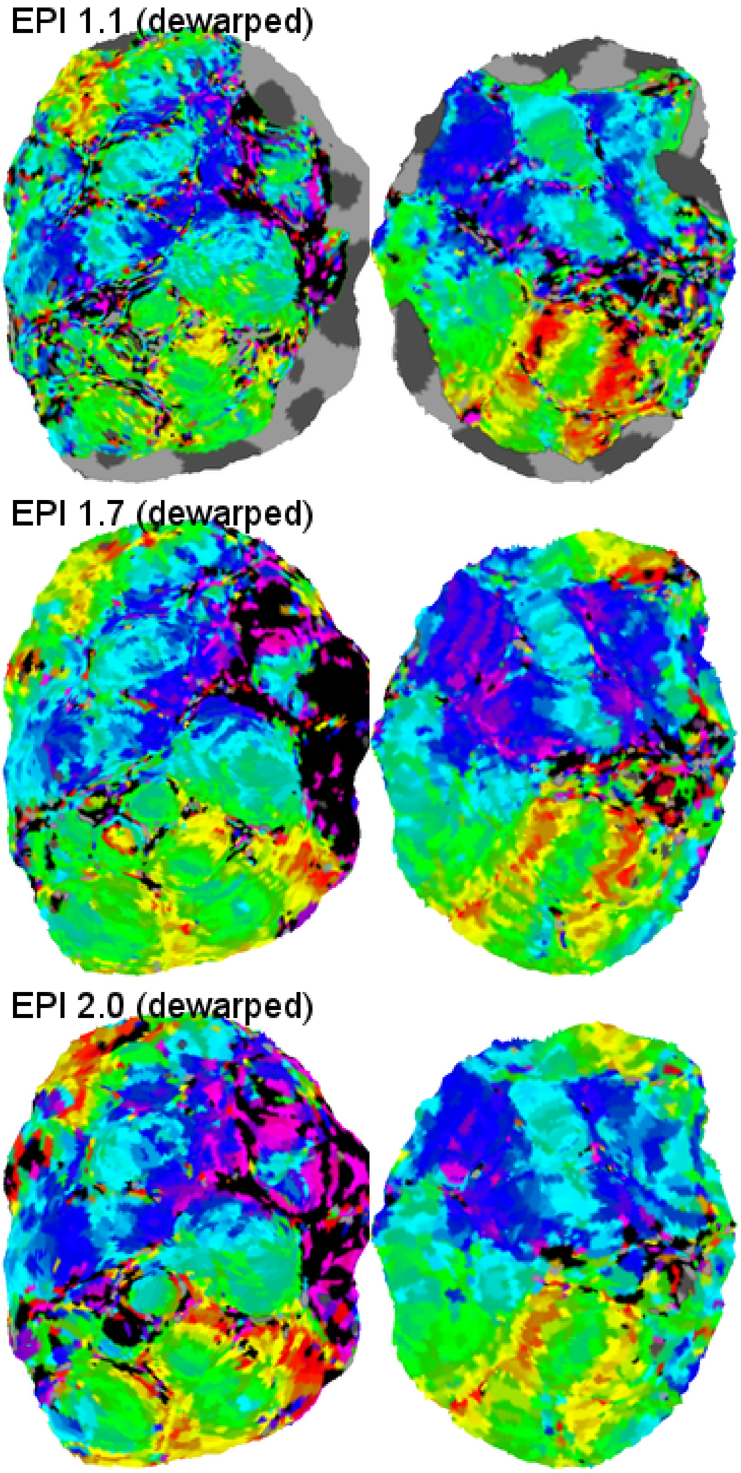

Supplementary Figure 11. Subject 4: EPI (dewarped)

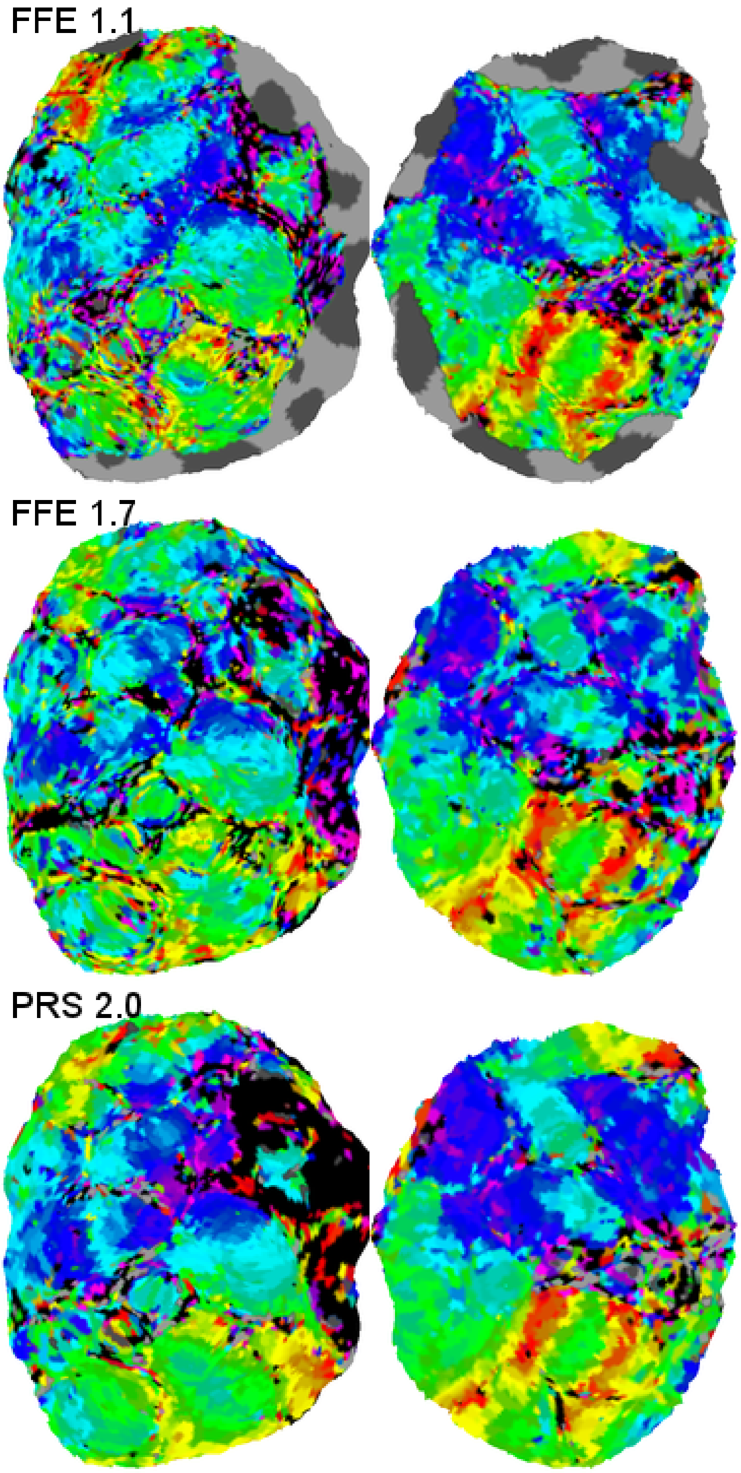

Supplementary Figure 12. Subject 4: FFE/PRESTO

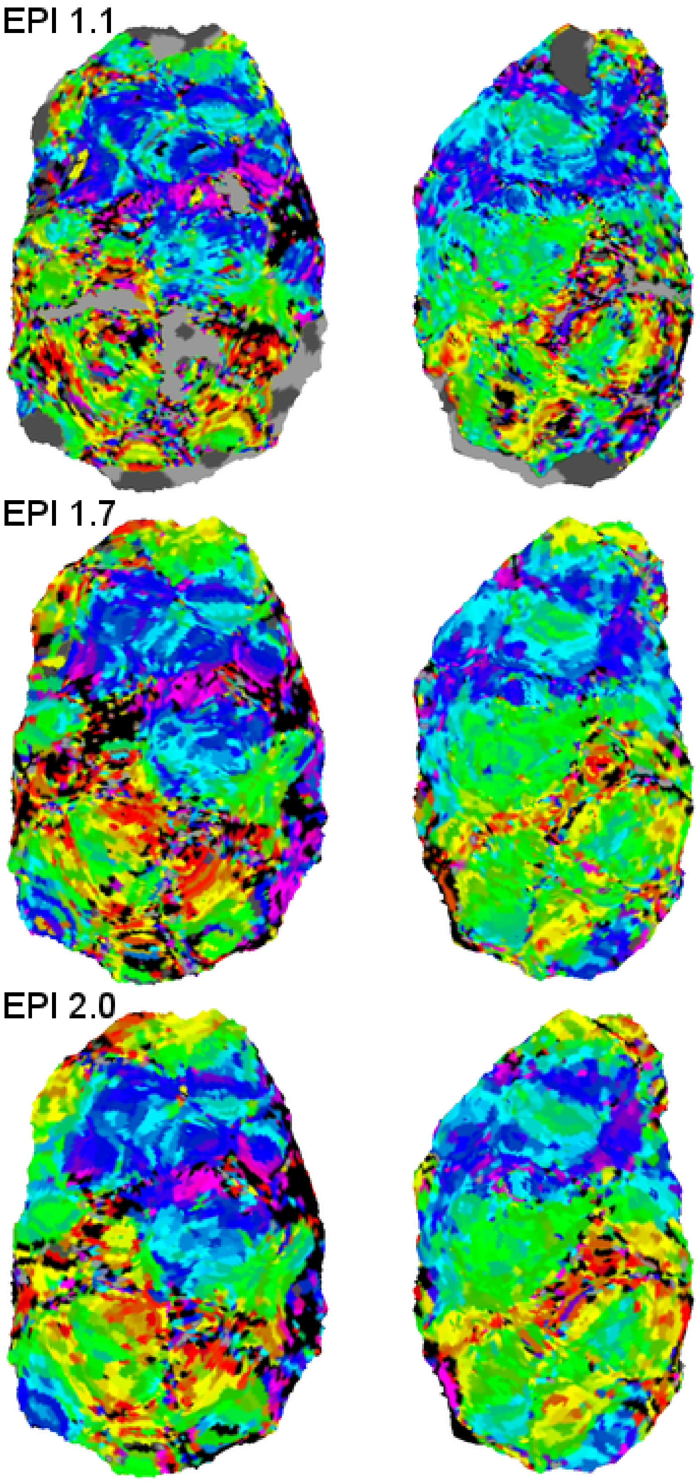

Supplementary Figure 13. Subject 5: EPI

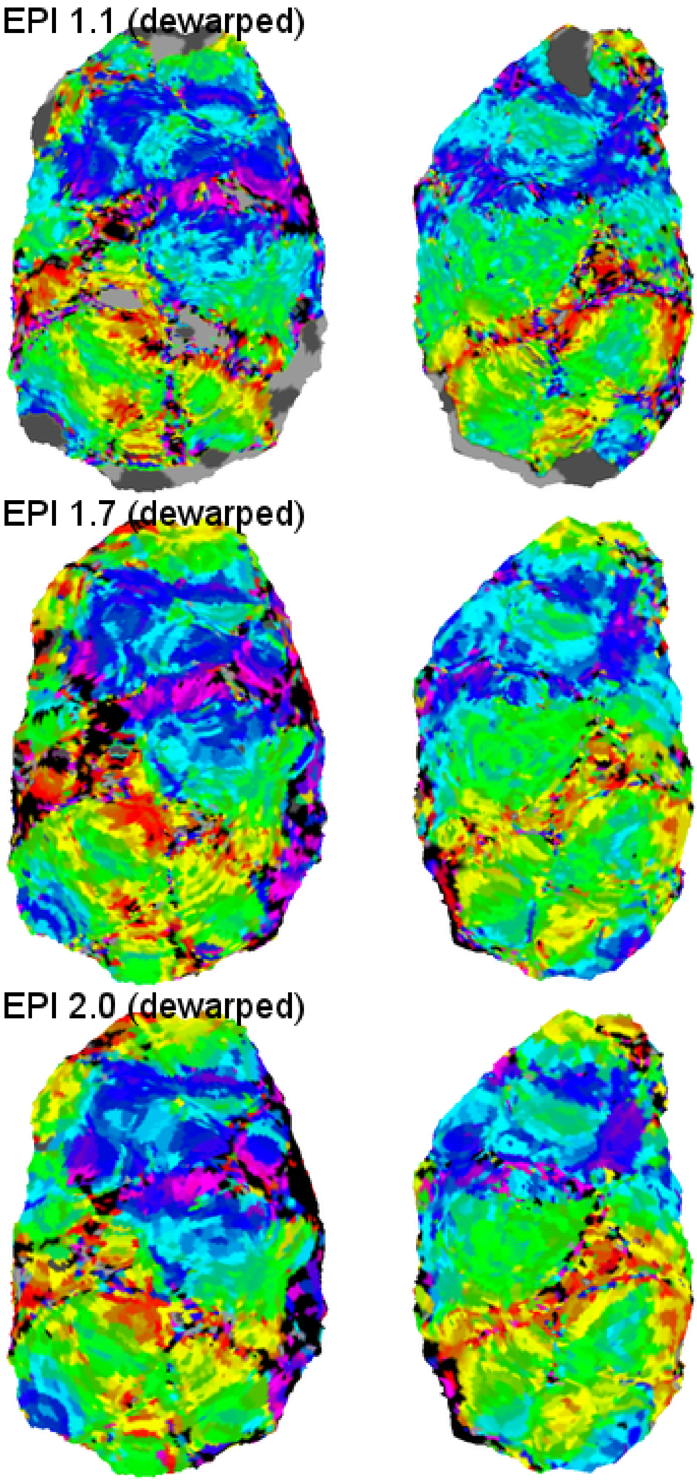

Supplementary Figure 14. Subject 5: EPI (dewarped)

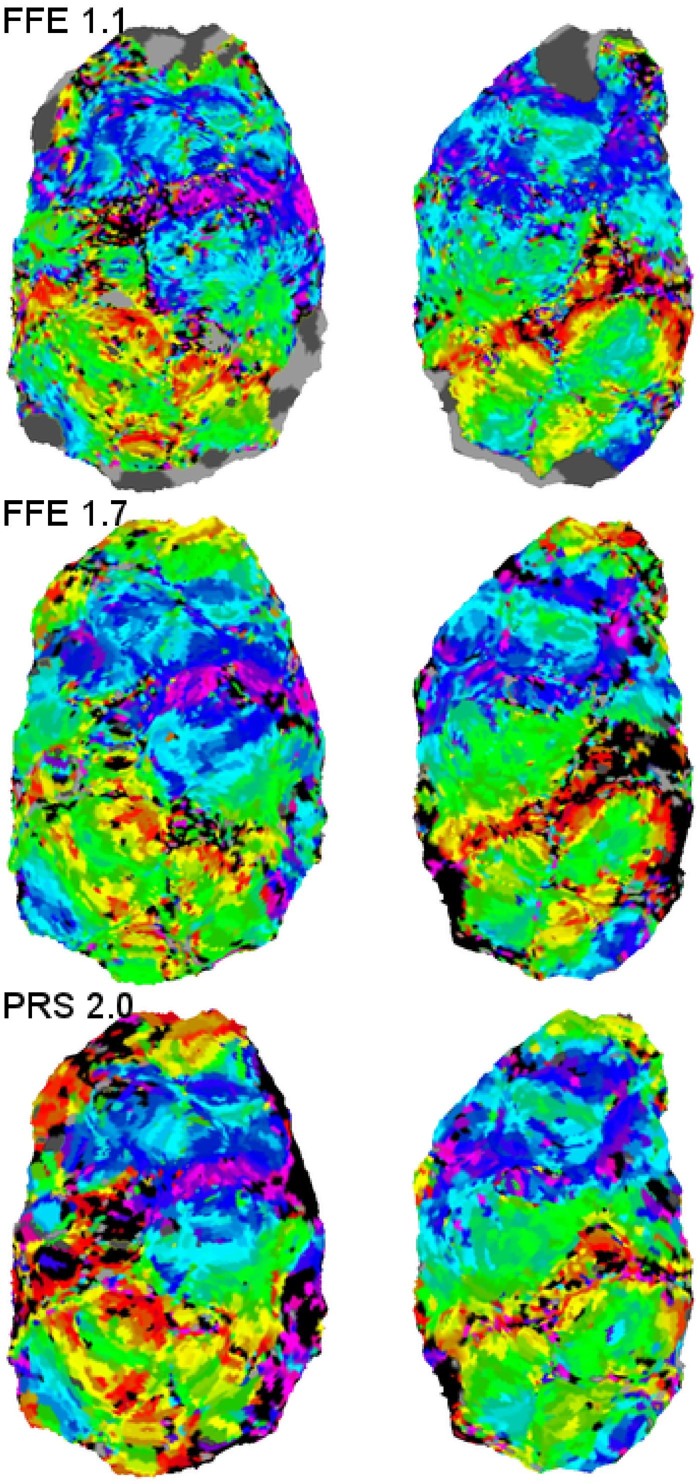

Supplementary Figure 15. Subject 5: FFE/PRESTO

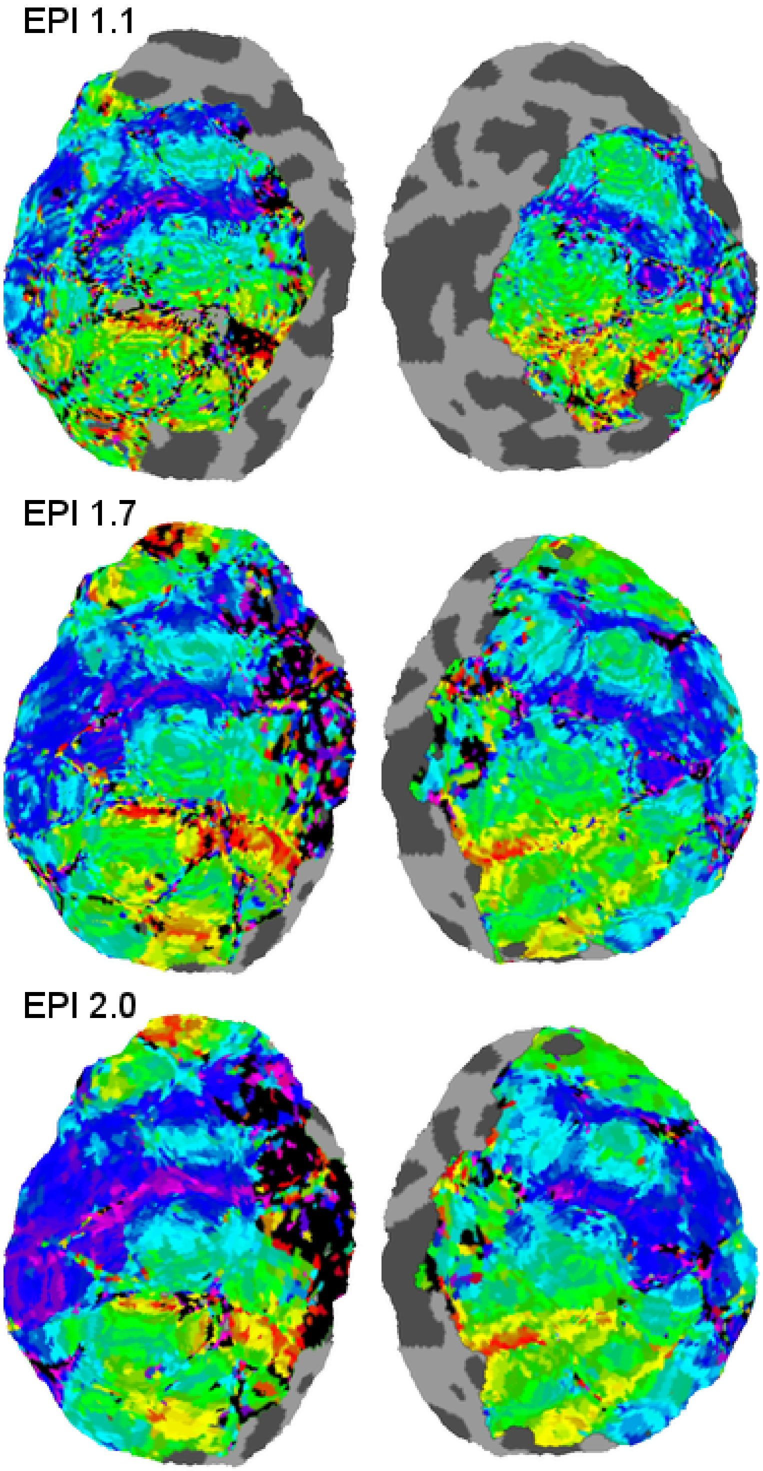

Supplementary Figure 16. Subject 6: EPI

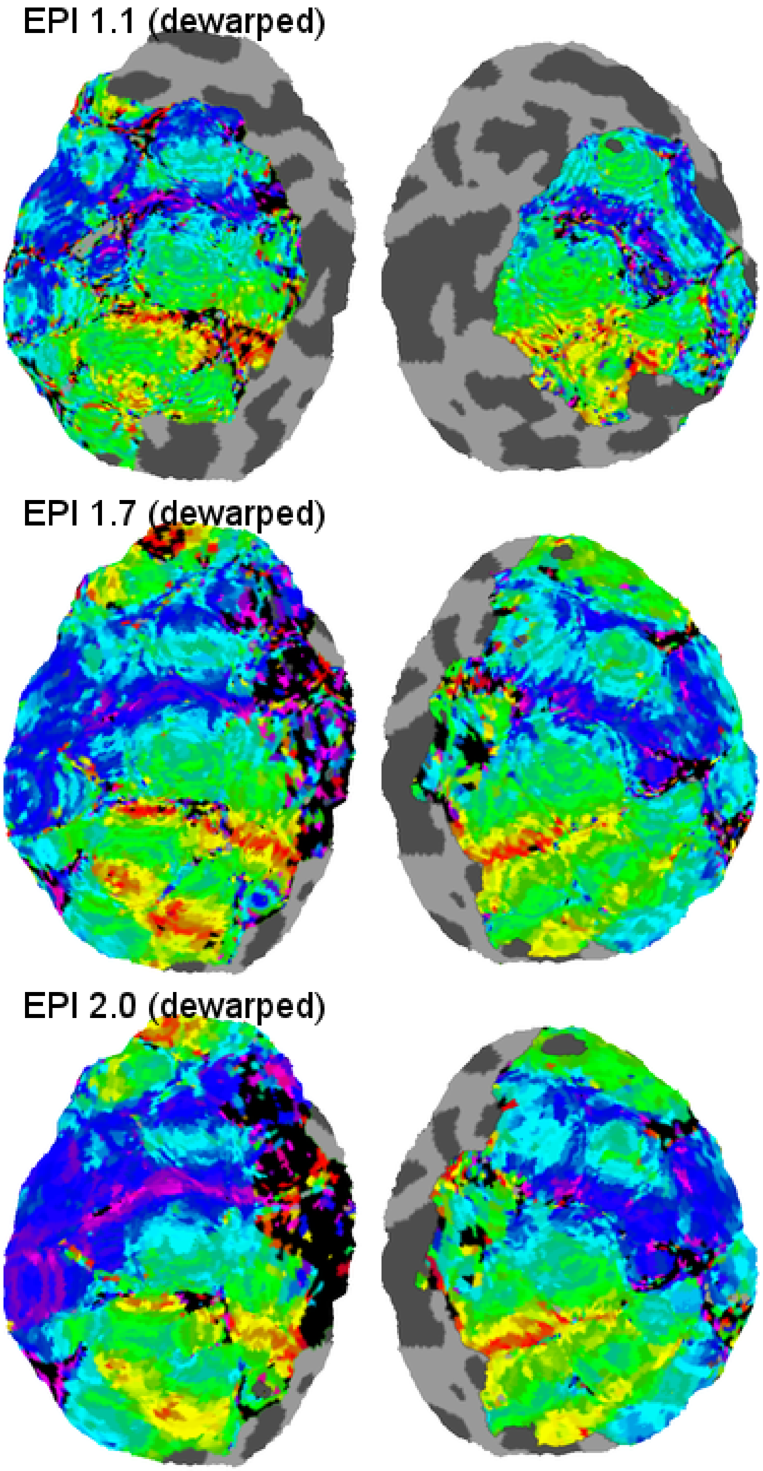

Supplementary Figure 17. Subject 6: EPI (dewarped)

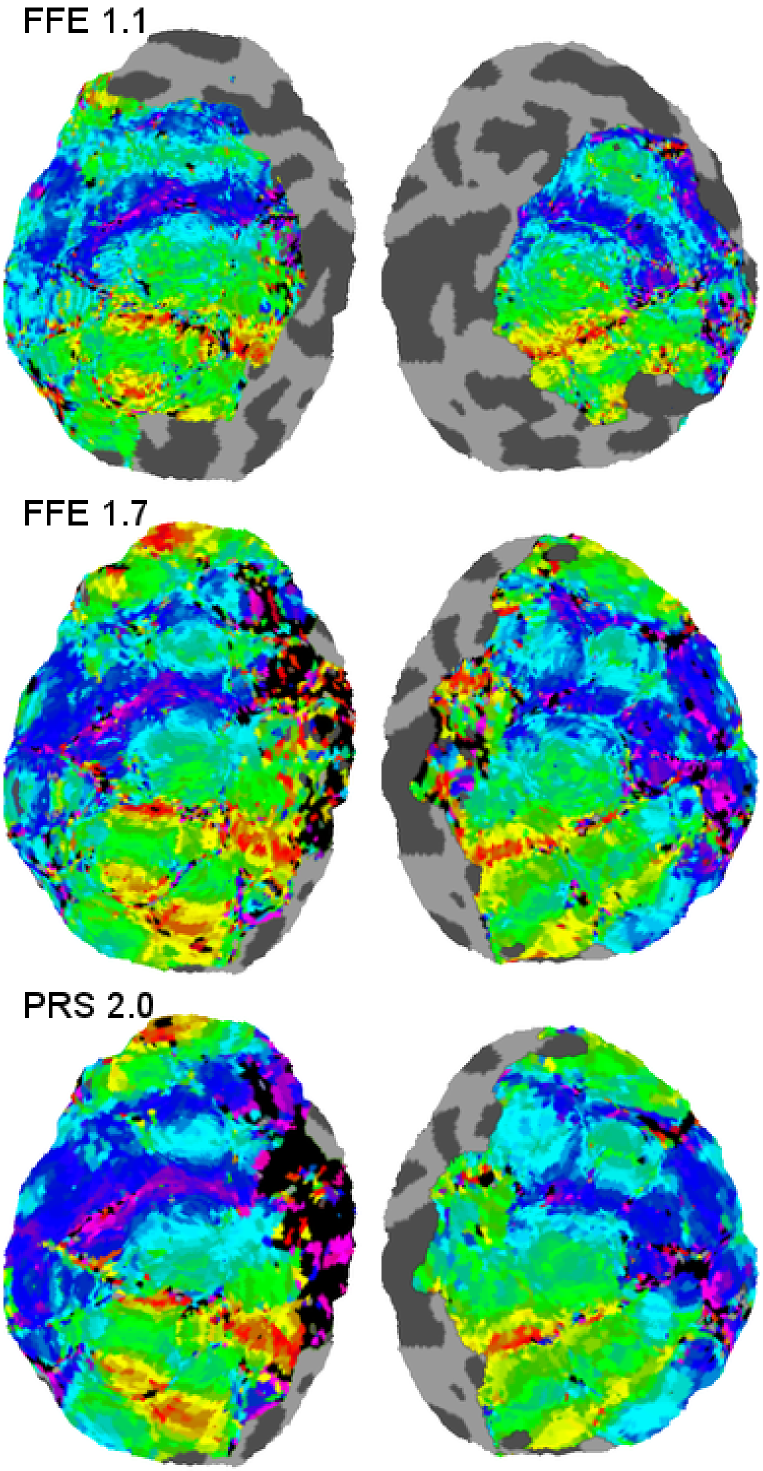

Supplementary Figure 18. Subject 6: FFE/PRESTO
