# Supplementary material for: Multishot versus Single-Shot Pulse Sequences in Very High Field fMRI: A Comparison Using Retinotopic Mapping
Source: PLoS One. 2012 Apr 13;7(4):e34626. doi: 10.1371/journal.pone.0034626 (PMC3326057; doi:10.1371/journal.pone.0034626)
Supplement: Information S2 — Representative mean, temporal noise, and tSNR images. Mean functional, noise standard deviation, and temporal signal to noise volume images are presented for the 1.12 mm EPI and FFE sequences in an example subject. (PDF) [file pone.0034626.s002.pdf]

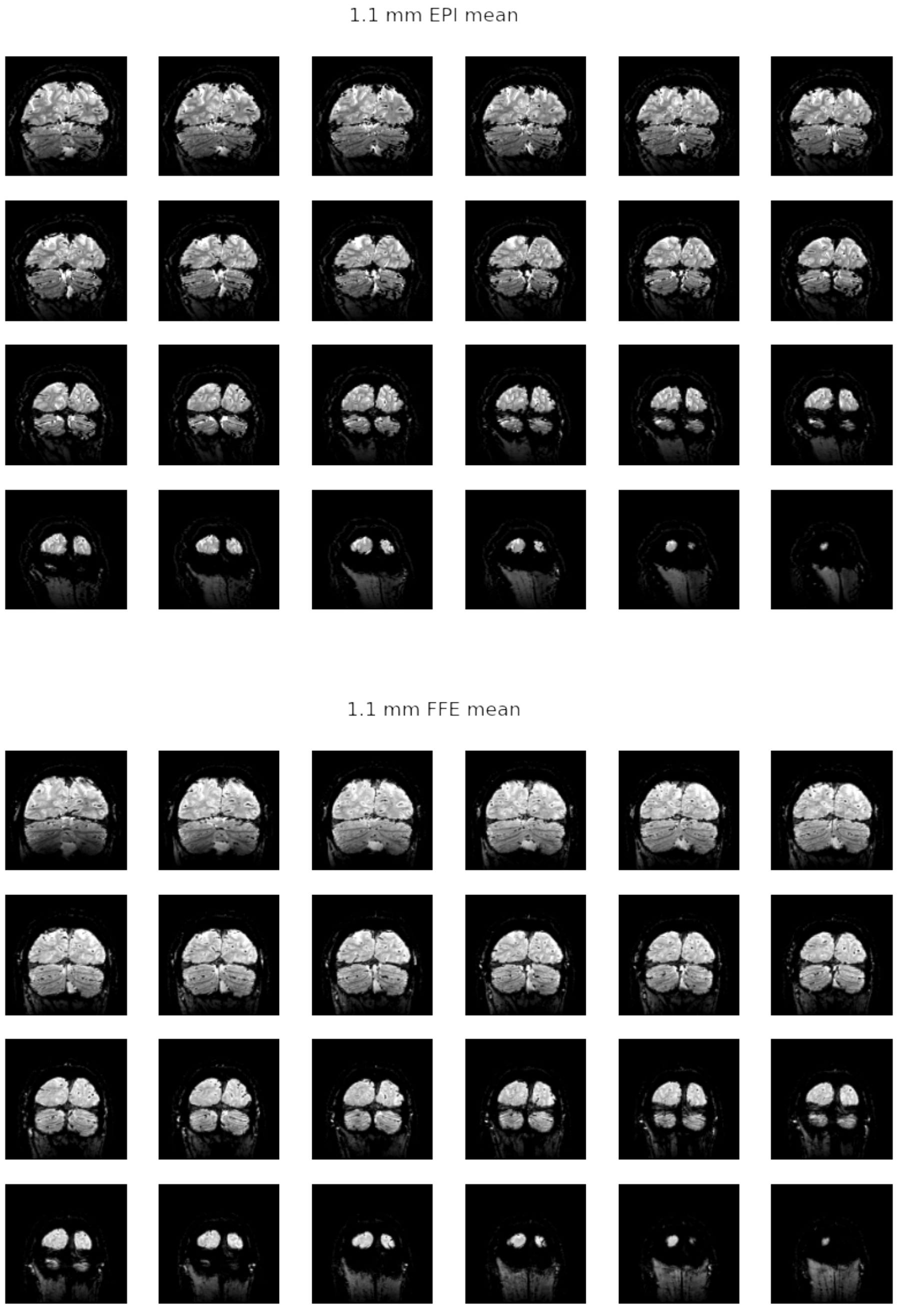

Supplementary Figure 1. Mean images for representative functional runs. To better illustrate the signal quality, these maps were calculated before motion correction or other preprocessing was applied (the analysis in the main text is performed on motion corrected data). No substantial Nyquist ghosting is evident in the images from either sequence type.

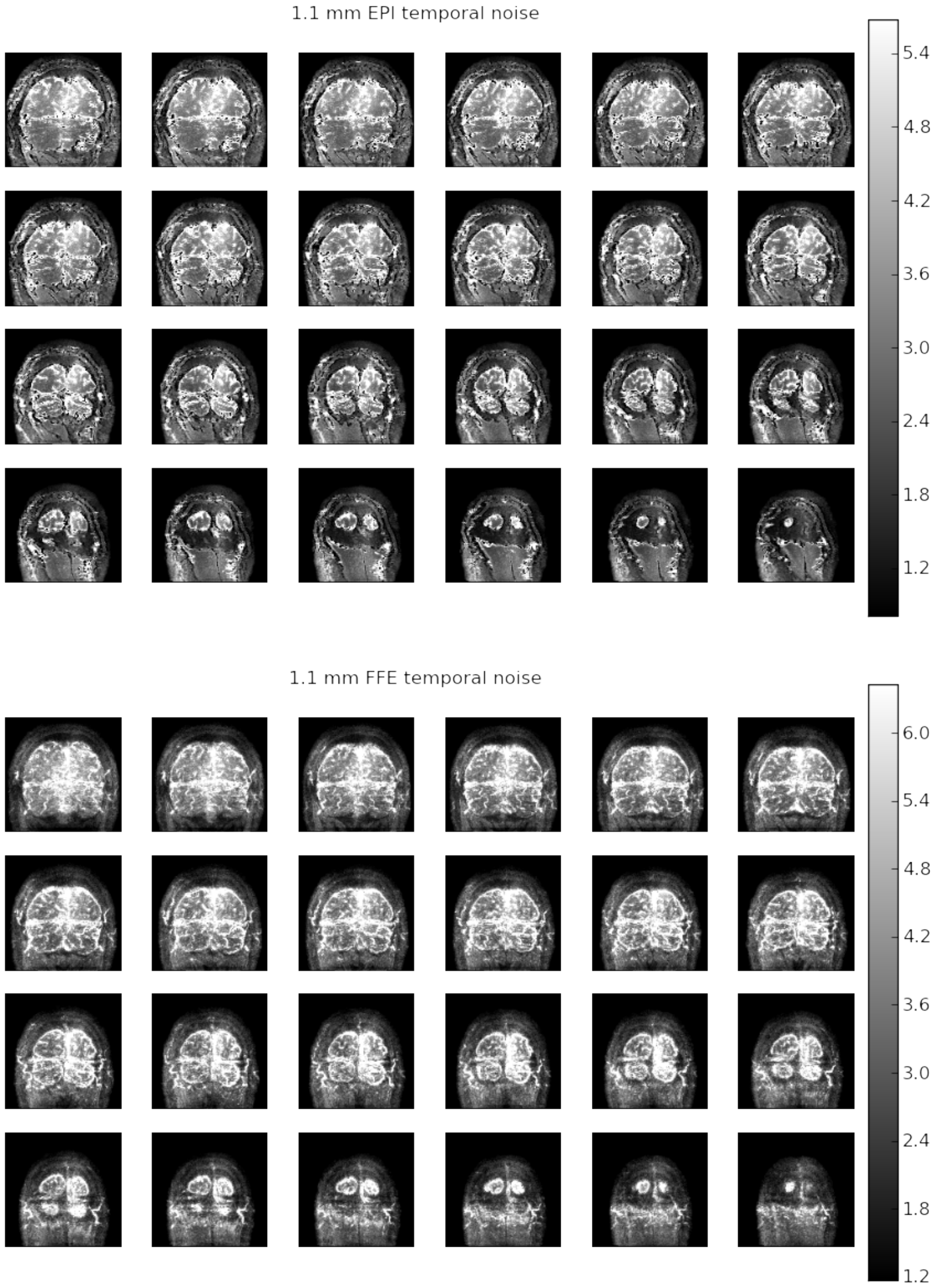

Supplementary Figure 2. Temporal noise maps for representative functional runs. Temporal noise is calculated as the standard deviation of the voxel time series. Apparent signals which originate well outside the head are suppressed during the SENSE reconstruction, resulting in a time course standard deviation of zero in these regions. The color scale is set so as to include the median 85% of values for each sequence.

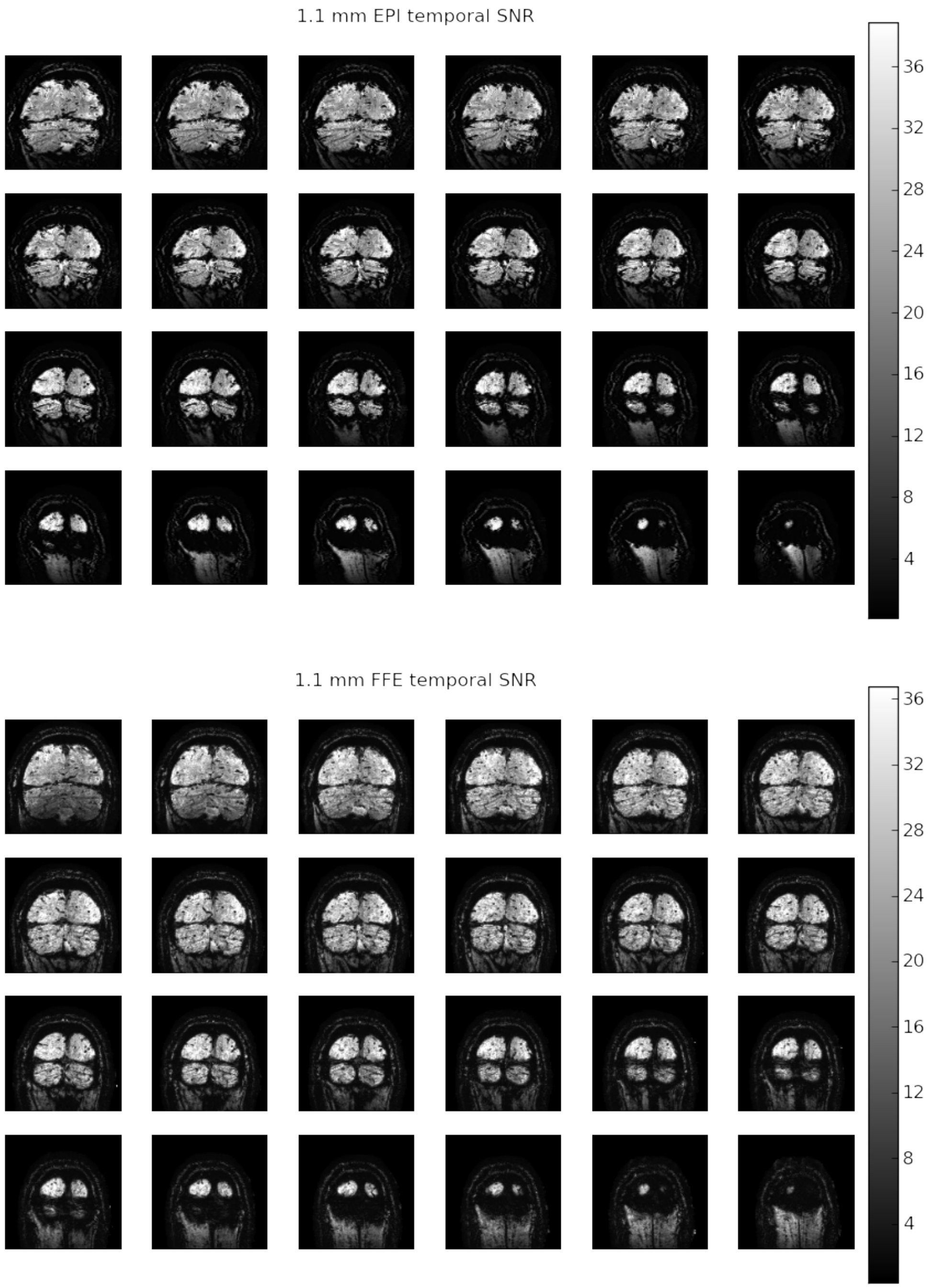

Supplementary Figure 3. Temporal SNR (tSNR) maps for representative functional runs. tSNR is calculated as the time series standard deviation (as in the previous figure) over the time series mean. The color scale here is set to include the median 98% of tSNR values for each sequence.
